# The *PHOSPHATE1* genes participate in salt and Pi signaling pathways and play adaptive roles during soybean evolution

**DOI:** 10.1186/s12870-019-1959-8

**Published:** 2019-08-14

**Authors:** Yan Wang, Huihui Gao, Lingli He, Weiwei Zhu, Lixin Yan, Qingshan Chen, Chaoying He

**Affiliations:** 1 State Key Laboratory of Systematic and Evolutionary Botany, Institute of Botany, Chinese Academy of Sciences, Nanxincun 20, Xiangshan, Beijing, 100093 China; 2 University of Chinese Academy of Sciences, Yuquan Road 19A, Beijing, 100049 China; 3 College of Agriculture, Northeast Agricultural University, Harbin, 150030 Heilongjiang China; 4 The Innovative Academy of Seed Design, Chinese Academy of Sciences, Beijing, 100101 China

**Keywords:** Abiotic stress, Adaptive evolution, Functional diversification, Gene expression, Orthologous gene, *PHO1* gene family, Soybean

## Abstract

**Background:**

The *PHOSPHATE1* (*PHO1*) gene family plays diverse roles in inorganic phosphate (Pi) transfer and signal transduction, and plant development. However, the functions and diversification of soybean *PHO1* family are poorly understood.

**Results:**

Cultivated soybean (*Glycine max)* was domesticated from wild soybean (*Glycine soja*). To illuminate their roles in this evolutionary process, we comparatively investigated the *G. max PHO1* genes (*GmPHO1*) in Suinong 14 (SN14) and *G. soja PHO1* genes (*GsPHO1*) in ZYD00006 (ZYD6). The sequences of the orthologous *Gm-GsPHO1* pairs were grouped into two Classes. The expression of Class I in both SN14 and ZYD6 was widely but relatively high in developing fruits, whereas Class II was predominantly expressed in the roots. The whole family displayed diverse response patterns to salt stresses and Pi-starvation in roots. Between SN14 and ZYD6, most *PHO1* genes responded similarly to salinity stresses, and half had sharp contrasts in response to Pi-starvation, which corroborated the differential response capacities to salinity and low-Pi stress between SN14 and ZYD6. Furthermore, in transgenic *Arabidopsis* plants, most Class II members and *GmPHO1;H9* from Class I could enhance salt tolerance, while only two Class II genes (*GmPHO1;H4* and *GmPHO1;H8*) differently altered sensitivity to Pi-starvation. The expression of critical genes was accordingly altered in either salt or Pi signaling pathways in transgenic *Arabidopsis* plants.

**Conclusions:**

Our work identifies some *PHO1* genes as promising genetic materials for soybean improvement, and suggests that expression variation is decisive to functional divergence of the orthologous *Gm-GsPHO1* pairs, which plays an adaptive role during soybean evolution.

**Electronic supplementary material:**

The online version of this article (10.1186/s12870-019-1959-8) contains supplementary material, which is available to authorized users.

## Background

Phosphorus is one of the most important macroelements for plants. Inorganic phosphate (Pi), the predominant form of phosphorus that is absorbed and utilized by plants, is limited in many soils. Under Pi-deficiency conditions, plants can remodel their root architecture to inhibit primary root growth and promote lateral root growth and root hair formation to improve phosphorus acquisition [1, 2]. Various Pi-starvation inducible genes involved in the Pi signaling pathway trigger different molecular processes to improve plant survival under Pi-starvation conditions [3], including *AT4*, *INDUCED BY PHOSPHATE STARVATION1* (*IPS1*), *PHOSPHATE TRANSPORTER* (*PHT1;4*), and *PHOSPHATE1* (*PHO1;H1*) in *Arabidopsis* [4–6]. *Arabidopsis PHO1* is the first gene that has been reported to be involved in the transfer of Pi from the roots to the shoots [7, 8], and in the signal transduction of Pi-deficiency response [9–11]. The *Arabidopsis pho1* mutant has low leaf Pi levels and shows severe shoot Pi-deficiency due to its defectiveness in loading Pi into the xylem [7]. *PHO1* is predominantly expressed in root stelar cells and participates in loading inorganic Pi into the xylem of roots [8]. The *PHO1*-like gene is involved in Pi transport in yeast (*Saccharomyces cerevisiae*) [12], suggesting a conserved role of *PHO1* in Pi transport and homeostasis.

The *Arabidopsis* genome contains 10 additional homologs of *PHO1* (identified as *PHO1;H1* to *PHO1;H10*), and their putatively encoded proteins contain SPX (named after SYG1, PHOSPHATASE 81, and XPR1) and EXS (named after ER RETENTION DEFECTIVE1, XPR1, and SYG1) domains [11, 12]. However, only *PHO1* and *PHO1;H1* are involved in Pi transport from the roots to the shoots [6]. Three *PHO1* homologs (*OsPHO1;1*, *OsPHO1;2*, and *OsPHO1;3*) have been identified in the rice genome, but only *OsPHO1;2* has been shown to play a role in loading Pi into the xylem [13]. These observations suggest that this gene family plays diverse roles. In corroboration with this notion, members of the *PHO1* gene family in *Arabidopsis* were also found to participate in signal transduction, as well as plant growth and development. *SHORT HYPOCOTYL UNDER BLUE1* (*SHB1*, also named as *PHO1;H4*) is involved in blue light signaling responses, flowering, and seed development [14–16], whereas *PHO1;H10* responds to various abiotic and biotic stresses and participates in the abscisic acid (ABA)-mediated signal transduction pathway [17]. Interestingly, *Arabidopsis PHO1* has also been reported to play an important role in stomatal responses to ABA [18], suggesting a possible interaction among different signal transduction pathways in plants such as drought and salinity stresses.

Salt stress largely influences global agriculture. Excessive soluble salts in soil can reduce plant growth, thereby severely affecting crop yield [19, 20]. Salt tolerance mechanisms are highly complex and can be controlled by the ionic signaling pathway, osmotic pressure, and the ABA-mediated pathway [21]. The salt overly sensitive (SOS) pathway controls the expression and activity of ion transporters by the SOS3-SOS2 protein kinase complex, which can be activated by salt stress-elicited calcium signals [21, 22]. The delta1-pyrroline-5-carboxylate synthetase gene (*P5CS1*), which encodes a rate-limiting enzyme involved in proline synthesis, can increase salt tolerance in plants by increasing osmotic pressure [23, 24]. Some other genes that are related to the ABA-mediated pathway such as the alcohol dehydrogenase gene (*ADH*) and *FIERY1* (*FRY1*) gene, are also involved in salt tolerance [25, 26]. These genes are often employed as marker genes in salt signaling pathways, and variations in the expression of these genes serve as diagnostic parameters in evaluating plant salt tolerance [21, 27].

Cultivated soybean (*Glycine max*) is thought to have been domesticated from wild soybean (*Glycine soja*) in China as early as 5000–9000 years ago [28, 29]. Various abiotic stresses largely limit soybean yield. The northeast region of China is a major soybean planting area, with a total of 3.73 × 10^6^ hm^2^ of sodic land [30]. Salt stress inhibits soybean development from germination to reproductive stage [31, 32] and sharply reduces grain yield to only 38.9% of that under normal conditions [33, 34]. Moreover, low availability of soluble Pi in the soil affects approximately half of the cultivated land around the world [35] and about 70% of the cultivated land is Pi-deficiency in China [36]. Pi-starvation significantly reduces grain yield by 40% in soybean, whereas Pi supply increases grain yield and pod number by 68 and 61%, respectively [37]. Several quantitative trait loci (QTLs) related to salinity tolerance and Pi-deficiency have been identified in soybean [38–42]. However, the mechanisms underlying the responses of soybean to these abiotic stresses remain unclear. Wild soybeans with wider geographic distributions and more genetic variations have adapted stress tolerance features compared to cultivated soybeans during evolution [43, 44]. Therefore, wild soybeans may serve as a rich germplasm for the improvement of cultivated soybeans. We previously identified 12 members of the *PHO1* gene family in cultivated soybeans and assessed their expression patterns in response to various stresses [45], which indicated their functions in abiotic stress tolerance. However, the functions of the *PHO1* gene family in soybean and functional diversification between wild and cultivated soybeans remain unclear. The present study compared the orthologous *PHO1* gene pairs between wild and cultivated soybeans in terms of sequence, tissue-specific expression, and responses to various abiotic stresses, including salinity and Pi-starvation conditions, followed by transgenic *Arabidopsis* analyses. This is the first investigation on the evolution and function of *PHO1* gene family in wild and cultivated soybeans. This study elucidates the adaptive roles of the *PHO1* gene family in soybean evolution, as well as reveals a potentially converged role of these genes in Pi and salinity signal pathways, thereby providing novel genetic material and ideas for crop improvement.

## Results

### Sequence comparison of the *GmPHO1* and *GsPHO1* cDNAs

**Fig. 1:**
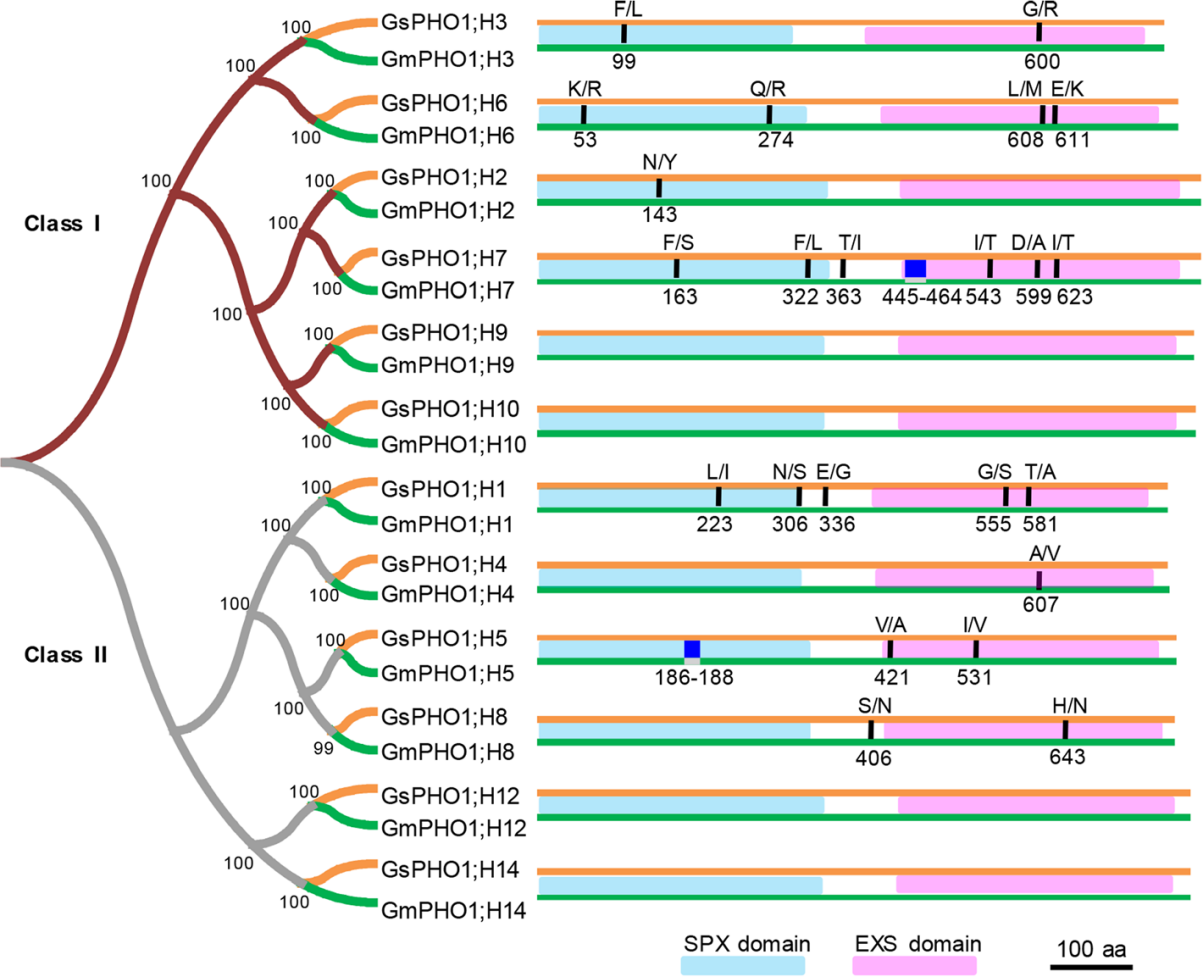
Sequence divergence of PHO1-like proteins from ZYD6 and SN14*.* The NJ phylogenetic tree was constructed based on PHO1 protein sequence of *G. soja* ZYD6 (orange lines) and *G. max* SN14 (green lines) respectively. The SPX and EXS domains are displayed in the blue and pink rectangles. The sequence variations (indels and substitutions) between each orthologous Gs-GmPHO1 pair are displayed by dark blue rectangles and dark vertical lines, respectively

Fourteen putative *PHO1* homologous genes were previously predicted [45], but 12 *PHO1*-like cDNAs were ultimately obtained from either the cultivated (prefix *Gm*) or wild (prefix *Gs*) soybeans (Additional file 1: Figures S1, S2). They were divided into Class Ι and Class II (Fig. 1, Additional file 1: Figure S2). When the *Arabidopsis PHO1* genes were included, we found that the functionally characterized *AtPHO1;H4* (*SHB1*) gene belonged to Class I, whereas *AtPHO1;H1* and *AtPHO1* were classified under Class II (Additional file 1: Figure S2), indicating functional divergence between Classes I and II. Moreover, each *PHO1* gene from cultivated soybean Suinong 14 (SN14) and wild soybean ZYD00006 (ZYD6) were clustered together to form an orthologous pair (Fig. 1, Additional file 1: Figure S2), suggesting that these originated from a common progenitor. The paralogs underwent distinct diversification during evolution, with differences ranging from 26.9 to 95.2% (Additional file 2: Table S1), suggesting functional divergence, whereas the sequence identities of the 12 orthologous pairs ranged from 99.3 to 100%. Two members (*PHO1*;*H9* and *PHO1*;*H10*) from Class I and two members (*PHO1*;*H12* and *PHO1*;*H14*) from Class II were identical between SN14 and ZYD6, and the remaining pairs contained few amino acid substitutions and indels (Fig. 1, Additional file 2: Table S2). We then estimated the potential effects of these sequence variations between the orthologous pairs by PROVEAN and SNAP analyses, which indicated that all amino acid substitutions and indels were neutral (Additional file 2: Table S2), suggesting that the functions of the corresponding orthologous gene pairs might be not altered. Each PHO1 orthologous pair from the cultivated and wild soybeans may thus likely have similar functions.

### Organ-specific expression of soybean *PHO1*-like genes

Assessment of expression diversity has been utilized to establish functional divergence among genes. To investigate the tissue-specific expression patterns of the *PHO1*-like genes, the total RNAs from the roots, leaves, stems, flowers, and developing fruits (1, 3, 5, and 15 days after pollination, shortened as fruit1, fruit3, fruit5, and fruit15) in SN14 and ZYD6 were subjected to qRT-PCR. Due to high sequence identity, common but specific primers were designed for paralogous gene pairs *PHO1;H1*/*H4*, *PHO1;H9/H10*, and *PHO1;H12/H14*. The results showed that the *PHO1* genes belonging to Classes I and II exhibited different organ-specific expression patterns (Additional file 1: Figure S3). Most soybean *PHO1* genes in Class Ι were differentially expressed among the vegetable and reproductive organs (Additional file 1: Figure S3a-e). A few extreme variations in expression were observed. *PHO1;H7* was active in most developing organs but hardly detected in roots (Additional file 1: Figure S3d), whereas *PHO1;H9*/*H10* were predominantly expressed in the roots (Additional file 1: Figure S3e). However, the expression patterns for genes in Class II were strikingly different from those in Class I. Soybean genes in Class II had similar expression patterns and were predominantly expressed in vegetable tissues, particularly the roots and stems (Additional file 1: Figure S3f-i). Moreover, the expression of these genes in fruits was relatively low (Additional file 1: Figure S3f-i).

Comparison of the orthologous *Gm-GsPHO1* pairs indicated that Class I genes displayed diverse expression patterns in various organs between SN14 and ZYD6. *GmPHO1;H7* had higher expression than *GsPHO1;H7* in all assessed organs (Additional file 1: Fig. S3d), whereas *GsPHO1;H3* had a relatively higher expression in flowers and early developing fruits (fruit1) than *GmPHO1;H3* in SN14 (Additional file 1: Figure S3b). Nevertheless, some genes such as *PHO1;H2* and *PHO1;H6* showed complex expression patterns in different tissues between SN14 and ZYD6. *GmPHO1;H2* exhibited lower expression levels in the roots, leaves, and flowers, but higher expression in other organs than *GsPHO1;H2* (Additional file 1: Figure S3a). Similarly, *PHO1;H6* showed lower expression levels in the roots and leaves but higher expression in the developing fruits in SN14 than in ZYD6 (Additional file 1: Figure S3c). Despite the complex expression patterns of genes in Class I, these showed relatively higher expression levels in developing fruits (fruit3-fruit15) in SN14 than in ZYD6. However, Class II genes were highly expressed in the vegetative plant parts in ZYD6 than in SN14 as well as in the roots, stem, or leaves (Additional file 1: Figure S3f-i).

**Fig. 2:**
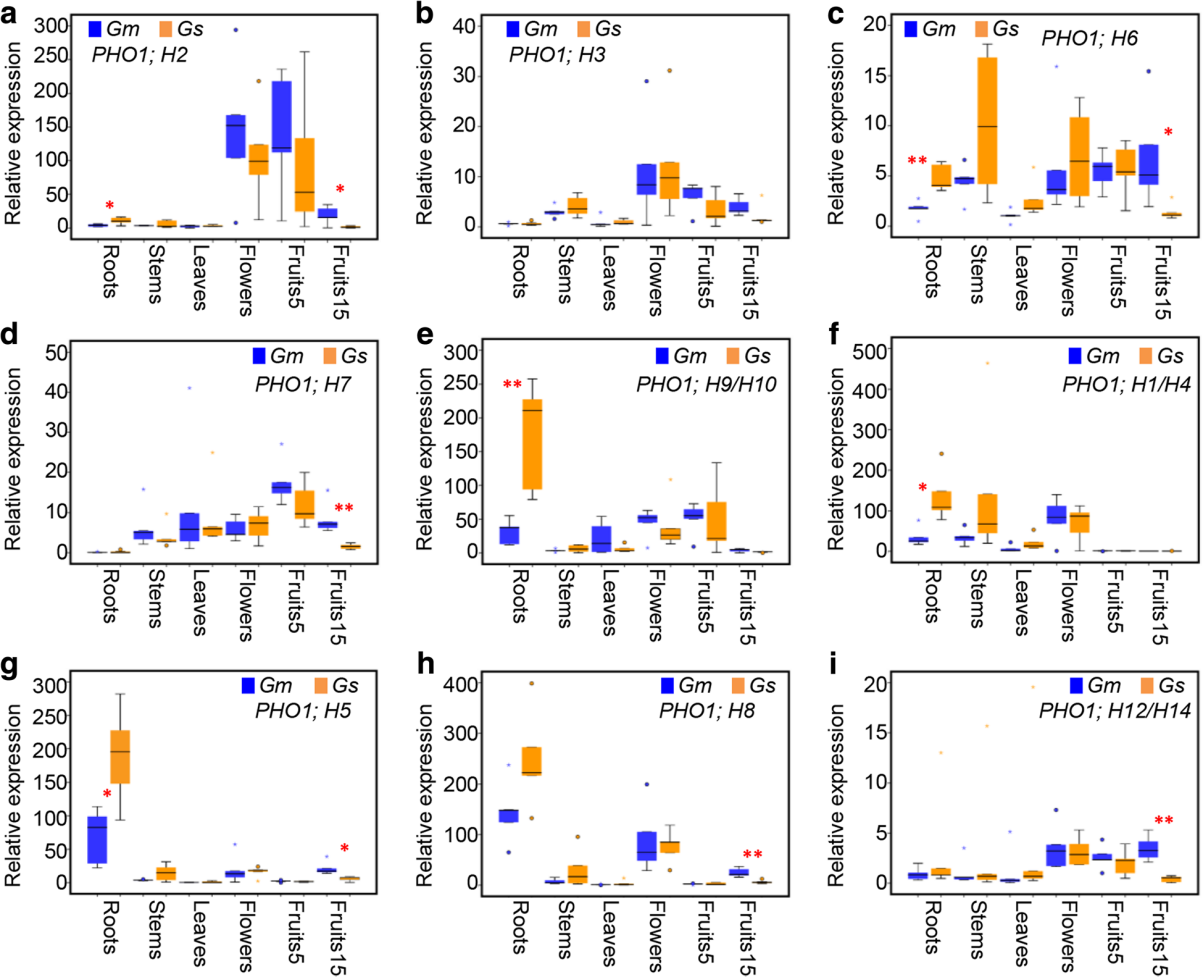
Variations in organ-specific expression of *PHO1* gene family in wild and cultivated soybeans. **a**-**e** Expression variation of soybean *PHO1* genes in Class I. **f**-**i** Expression variation of soybean *PHO1* genes in Class II. *G. max* (*Gm*) includes five cultivars (SN14, HF, NF, HN, and DN), and *G. soja* (*Gs*) consists of five wild accessions (ZYD6, Y1, Y2, Y3, and Y4). The gene expressions in various tissues as indicated were analyzed, and the raw data are shown in Additional file 1: Figures S3 and S4

The above observations between ZYD6 and SN14 were further verified by qRT-PCR analyses using additional four wild (Y1, Y2, Y3, and Y4) and four cultivated (Hefeng48, Nenfeng16, Heinong35, and Dongnong53) soybeans (Additional file 1: Figure S4). Despite few fluctuations, these reflected similar expression patterns in different tissues in ZYD6 and SN14. Taken together, the integrated analysis and main findings indicated that both Classes I and II exhibited conserved tissue-specific expression patterns either in wild or cultivated soybeans: Class I had a broader expression domains than Class II, in which most genes were predominantly expressed in the roots (Fig. 2). Interestingly, genes that had differential expression patterns in the roots between wild and cultivated soybeans had higher expression in the roots of wild soybeans than cultivated ones, whereas genes that were differentially expressed in fruit15 between *G. max* and *G. soja* had higher expression in fruit15 of cultivated soybeans than wild ones (Fig. 2), suggesting functional divergence of Classes I and II *PHO1* genes in fruit (seed) development and stress responses.

### Soybean *PHO1*-like gene expression under various stresses

#### Salinity stresses

**Fig. 3:**
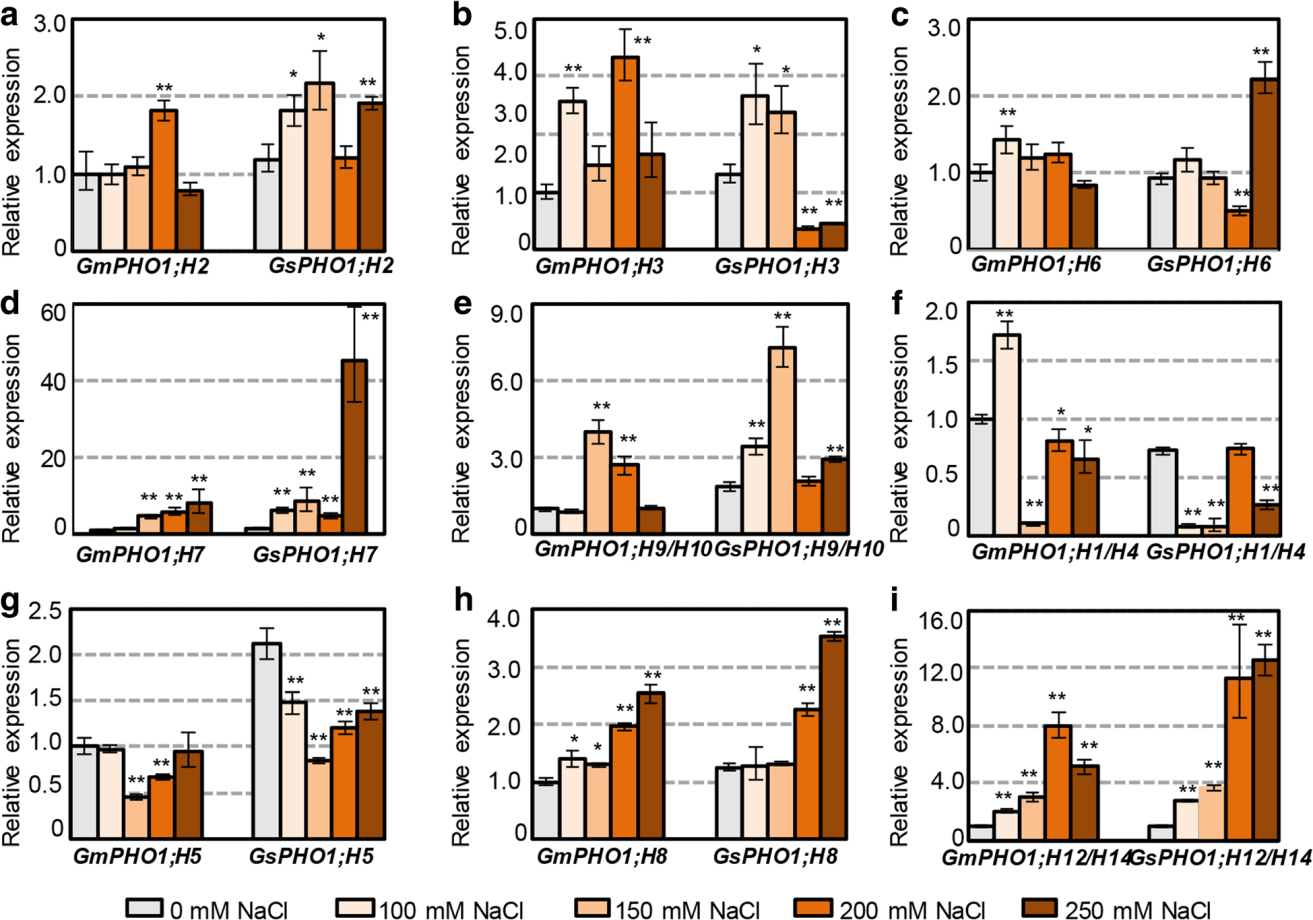
Expression of soybean *PHO1* genes in response to salt stress. **a**-**e** The expression of *Gm*-*GsPHO1* orthologous gene pairs in Class I. **f**-**i** The expression of *Gm*-*GsPHO1* orthologous gene pairs in Class II. Total RNAs from roots of two-week old seedlings of ZYD6 and SN14 under salt stresses with different concentration of NaCl for 4 h were subjected to qRT-PCR. Expression of each gene in the non-treated conditions (gray column) was used as control. For ease in comparison of *Gm*-*GsPHO1* orthologous gene pairs, the *GmPHO1* expression was set as 1 under the untreated conditions. The * means significance at the *P* < 0.05 level, and the ** represent the significance at the *P* < 0.01 level

To further illustrate the diversity of soybean *PHO1*-like genes in response to abiotic stresses, two-week-old seedlings of SN14 and ZYD6 were treated with different levels of salinity, and total RNA from the roots were subjected to qRT-PCR analysis (Fig. 3). The Class I genes *PHO1;H2*, *PHO1;H7*, and *PHO1;H9*/*H10* and the Class II genes *PHO1;H5*, *PHO1;H8*, and *PHO1;H12*/*H14* showed similar response patterns to salt stress between SN14 and ZYD6 (Fig. 3a, d-e g-i), whereas the remaining genes, including *PHO1;H3* and *PHO1;H6* in Class I and *PHO1;H1*/*H4* from Class II, displayed relatively different expression patterns under salt stress between SN14 and ZYD6 (Fig. 3b-c, f). The *Gm*-*GsPHO1;H12*/*H14* orthologous pairs were all consistently induced under all the concentration of salt stress with the stronger response in ZYD6. Although some orthologous pairs showed similar expression patterns under salt stress, one or two genes of the pairs only responded to certain concentrations such as *PHO1;H5* and *PHO1;H8* in Class II and the Class I members of *PHO1;H2* and *PHO1;H9*/*H10* (Fig. 3a, e, g-h). The expression of *GmPHO1;H5* decreased only at 150 mM and 200 mM NaCl, but *GsPHO1;H5* expression decrease at all concentrations. *GmPHO1;H8* expression was induced by salt stress, whereas that of *GsPHO1;H8* only increased with 200 mM and 250 mM NaCl treatment. Furthermore, paralogs of the *Gs*-*GmPHO1* genes showed variations in both tendency and magnitude of gene expression. These findings indicate that the soybean *PHO1* genes may have diverse roles in salt stress responses.

#### Low-Pi treatments

**Fig. 4:**
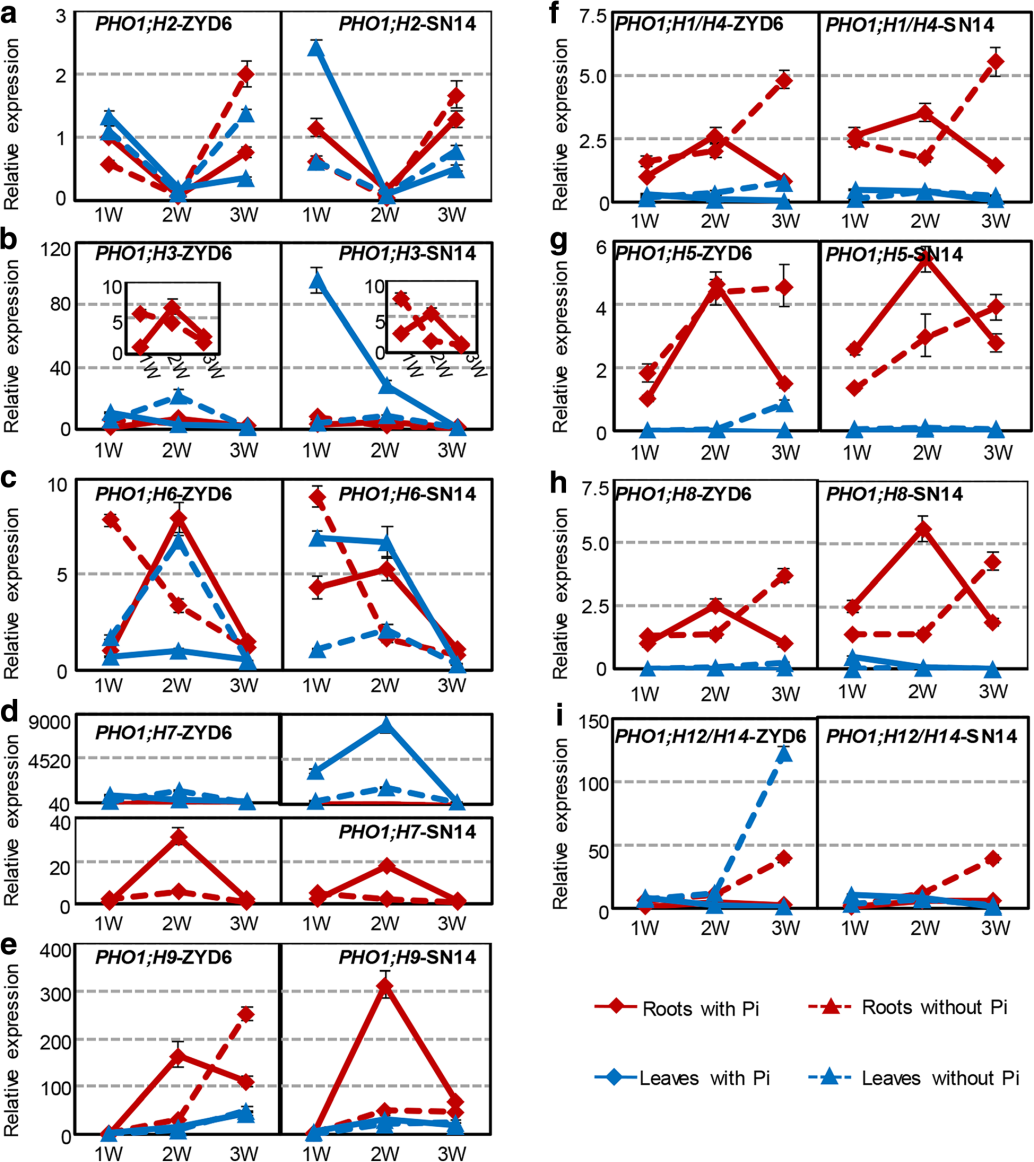
Expression of soybean *PHO1* genes in response to Pi-deficiency. **a**-**e** The expression of *PHO1* orthologous genes from Class I in ZYD6 and SN14. **f**-**i** The expression of *PHO1* orthologous genes from Class II in ZYD6 and SN14. The soybeans were grown in normal (1.25 mM Pi) and Pi-deficiency (−Pi, 0 mM Pi) Hogland medium. Gene expression in the roots and leaves was detected by qRT-PCR under Pi-deficiency for one, two and three weeks respectively. Expression of each gene in the normal conditions at 1 week in the roots of ZYD6 was set to 1. The * means significance at a *P* < 0.05 level, and the ** represent the significance at a *P* < 0.01 level. W, week

We also investigated the expression of the *PHO1* genes in soybean roots and leaves under Pi-starvation (Fig. 4). Soybean *PHO1* genes in Class I exhibited diverse expression profiles under normal Pi conditions (solid lines in Fig. 4a-e) and these showed complex responses to Pi-deficiency in the roots and leaves (dashed lines in Fig. 4a-e). However, the orthologous gene pairs of *PHO1;H2*, *PHO1;H3*, *PHO1;H6*, and *PHO1;H7* showed similar responses to low-Pi treatments in both roots and leaves between ZYD6 and SN14, whereas *PHO1;H9/10* depicted a similar expression pattern only in the leaves between ZYD6 and SN14. Similar *PHO1;H9/10* expression patterns were observed in the roots of ZYD6 and SN14 in the first 2 weeks after low-Pi treatments; however, upregulation was observed in ZYD6 under Pi stress at the third week (Fig. 4e). In Class I, only *PHO1;H9/10* was significantly and continuously upregulated in the roots of ZYD6 under Pi-starvation, suggesting its roles in response to low-Pi conditions.

Class II *PHO1* genes (*PHO1;H1/4*, *PHO1;H5*, *PHO1;H8*, and *PHO1;H12/14*) showed similar expression profiles during root and leaf development under normal conditions (red and blue solid lines, Fig. 4f-i). With Pi-deficiency, these were similarly upregulated in the roots between ZYD6 and SN14 and reached a maximum at 3 weeks (red dashed lines in Fig. 4f-i). These genes were upregulated in the leaves of ZYD6, whereas their expression in the leaves of SN14 did not change (dashed blue lines in Fig. 4f-i).

These findings suggest that the *PHO1* gene family may play roles in soybean adaptation and morphological divergence. Moreover, the expression variation is likely decisive evolutionary event between the orthologous pairs of *PHO1* genes, which occurred during the divergence of wild and cultivated soybeans. We therefore functionally tested these assumptions by overexpressing the cDNA of these genes from cultivated soybean in transgenic *Arabidopsis*.

### Overexpressing *GmPHO1* genes in transgenic *Arabidopsis*

Due to the highly identical sequences within paralog pairs *GmPHO1;H9*/*H10* and *GmPHO1;H12/H14* (Additional file 2: Table S1), the *GmPHO1;H10* and *GmPHO1;H14* were excluded, and thus altogether 10 *GmPHO1* genes from the SN14 (cultivar) were included in the transgenic *Arabidopsis* assays. Three T_3_ lines of each transgene that were confirmed via RT-PCR (Additional file 1: Figure S5) were further investigated, and all obtained *GmPHO1* transgenic plants showed no obvious phenotypic variations compared to wild-type (WT) *Arabidopsis*, including flowering time, seed size, plant height, and germination rate under normal growth conditions (Additional file 2: Table S3), indicating that overexpressing *GmPHO1* did not affect plant development.

#### GmPHO1 altered the salt tolerance of transgenic Arabidopsis plants

**Fig. 5:**
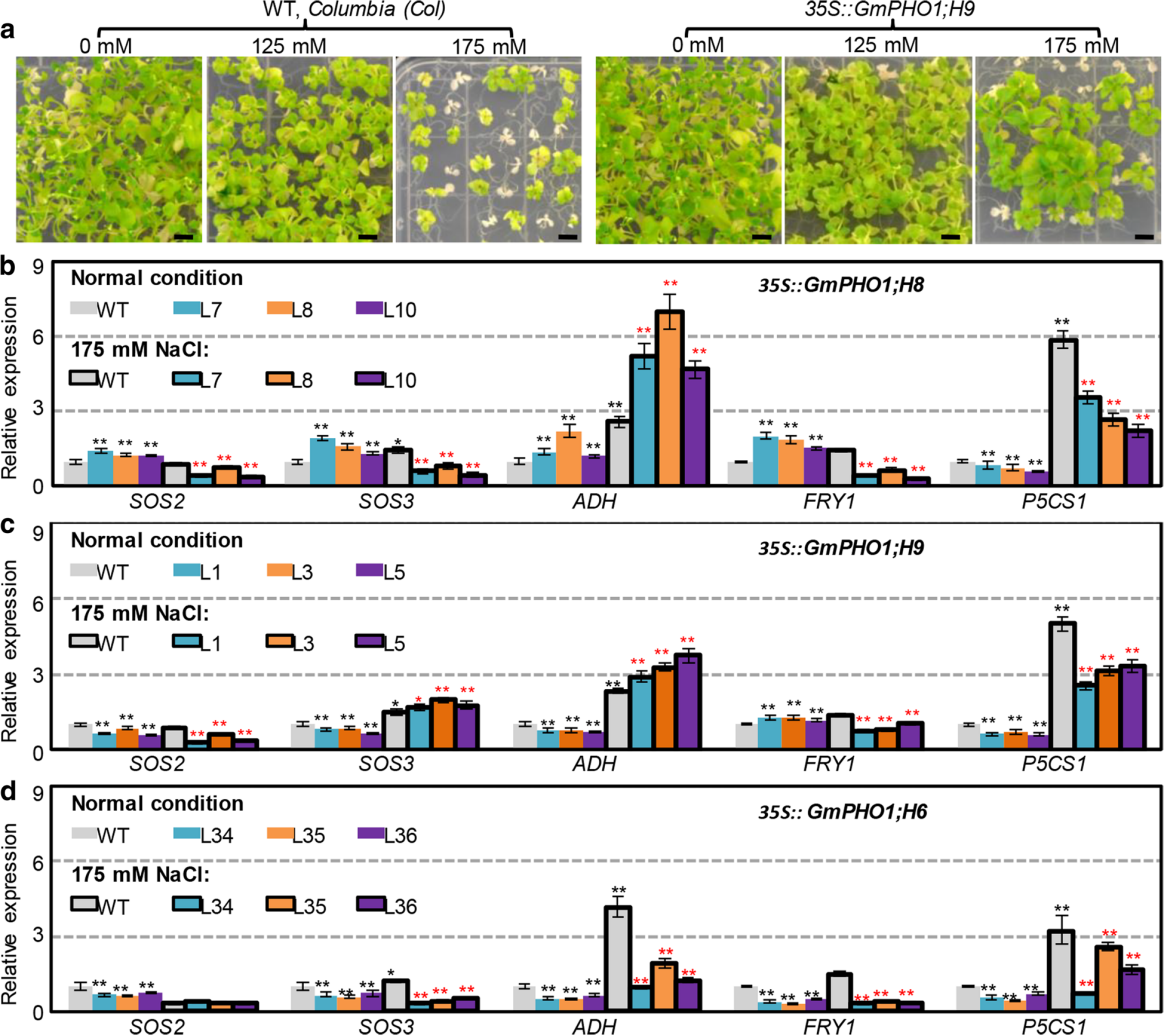
*GmPHO1* altered the salt tolerance of transgenic *Arabidopsis* plants. **a** The growth of *GmPHO1;H9* transgenic plants under different salt stress. Wild-type (WT) of *Arabidopsis* (Col) was used as controls. The seedlings growing in medium for 6 weeks were captured. Bar = 0.5 cm. **b-d** Expression of salt tolerance-related genes in transgenic *Arabidopsis*. The expression of *SOS2*, *SOS3*, *ADH*, *P5CS1* and *FRY1* in transgenic *Arabidopsis* plants harboring *GmPHO1;H8* (**b**), *GmPHO1;H9* (**c**) and *GmPHO1;H6* (**d**), respectively. WT and transgenic lines were grown in 1/2 MS medium for 1 week and then transferred to the salt medium with 175 mM NaCl for 24 h. Expressions of each gene in WT (gray column) under 0 mM NaCl were set as 1. The black stars (* or **) indicate the difference significance relative to the gene expression in WT under 0 mM NaCl, whereas the red ones indicate a significant difference relative to the gene expression in WT under 175 mM NaCl. The * means significance at the *P* < 0.05 level, and the ** represent the significance at the *P* < 0.01 level

Because alterations in the expression of soybean *PHO1* genes occur in response to salinity stresses, we first evaluated the behavior of the *GmPHO1* transgenic plants under salinity stresses (Fig. 5, Additional file 1: Figure S6). The seed germination and seedling green rate were evaluated under salt stresses (125 and 175 mM NaCl, respectively). The germination rates of transgenic lines were all higher than those of WT under 125 mM NaCl for 2 days; however, all included plant lines could germinate normally with increasing treatment time (Additional file 2: Table S4). Similar patterns were observed with 175 mM NaCl treatment (Additional file 2: Table S4). These results indicate that transgenes could enhance seed germination speed.

No significant difference in the green rate of seedlings was observed under 125 mM NaCl conditions; however, differences were observed under 175 mM NaCl conditions (Additional file 1: Figure S6a). Only *35S::GmPHO1;H9* from Class I transgenic plants showed significantly higher seedling green rate than WT under NaCl stress (Fig. 5a, Additional file 1: Figure S6a). However, the *35S::GmPHO1;H1*, *35S::GmPHO1;H5*, 3*5S:*:*GmPHO1;H8*, and *35S::GmPHO1;H12* transgenic lines of Class II exhibited a higher seedling green rate than WT under 175 mM NaCl stress (Additional file 1: Figure S6a). These observations show that *GmPHO1* enhances the salt tolerance, however, the function is obviously diverged between Class I and Class II since only one gene in Class I and many in Class II could confer salt tolerance to transgenic *Arabidopsis* plants.

Transgenic plants of *35S::GmPHO1;H6*, *35S::GmPHO1;H8*, and *35S::GmPHO1;H9* were selected for the evaluation of salt tolerance in the soil. The four-week old seedlings were supplied with 250 mM NaCl to induce salt stress. After 15 days of treatment, the WT and transgenic lines harboring *GmPHO1;H6* showed extensive bleaching and wilting, whereas the *35S::GmPHO1;H8* and *35S::GmPHO1;H9* transgenic lines all exhibited intense green color and were more vigorous than the WT (Additional file 1: Figure S6b-e). Furthermore, *35S::GmPHO1;H8* and *35S::GmPHO1;H9* transgenic plants all showed significantly higher chlorophyll content than the WT and the *35S::GmPHO1;H6* transgenic lines (Additional file 1: Figure S6f). Moreover, the aboveground biomass of *35S::GmPHO1;H8* and *35S::GmPHO1;H9* transgenic plants after one-month treatment were much higher than the WT and *35S::GmPHO1;H6* lines (Additional file 1: Figure S6 g). These observations indicate that overexpressing soybean *PHO1* genes (*GmPHO1;H1*, *GmPHO1;H5*, *GmPHO1;H8*, *GmPHO1;H9*, and *GmPHO1;H12*) improves salt tolerance in *Arabidopsis*.

#### GmPHO1 affects the salt tolerance pathways in transgenic Arabidopsis

The *SOS2*, *SOS3*, *ADH*, *P5CS1*, and *FRY1* genes are essentially involved in salt tolerance pathways in plants [21–26]. To elucidate the possible connection of *GmPHO1* with salt stress pathways, we checked the expression of these salt tolerance pathway genes in transgenic plants. The seven-day-old seedlings of WT, *35S::GmPHO1;H8*, *35S::GmPHO1;H9*, and *35S::GmPHO1;H6* transgenic *Arabidopsis* plants were transferred to a medium with 175 mM NaCl for 24 h, and total RNA from the whole seedlings was subjected to qRT-PCR. Under normal growth (0 mM NaCl) conditions, we found that each transgene could differentially affect the expression of these marker genes compared to the WT (highlighted by the black stars above the open color columns in Fig. 5b-d). Furthermore, we observed that *ADH*, *P5CS1*, *SOS3*, and *FRY1* were upregulated by NaCl treatment, whereas *SOS2* expression was not significantly affected in the WT background (highlighted by the black star above the blocked gray columns compared to the corresponding open gray columns in Fig. 5b-d). Moreover, these salt pathway marker genes were differentially expressed in the transgenic plants compared to the WT under salt stress conditions (highlighted by the red stars above the blocked color columns relative to the blocked gray column in Fig. 5b-d).

In the *35S::GmPHO1;H8* transgenic plants, except for the *P5CS1* downregulation, the *SOS2*, *SOS3*, *ADH,* and *FRY1* genes were upregulated under 0 mM NaCl. However, compared to the WT under salt treatments, only the expression of *ADH* sharply increased, whereas the other genes were downregulated (Fig. 5b). Nonetheless, relative to the corresponding transgenic lines under 0 mM NaCl, both *ADH* and *P5CS1* were upregulated, and the other genes were downregulated under salinity stress (Fig. 5b)*.* Compared to the WT, in *35S::GmPHO1;H9* transgenic *Arabidopsis*, *SOS2*, *SOS3*, *ADH* and *P5CS1* were downregulated, whereas *FRY1* was upregulated under 0 mM NaCl conditions (Fig. 5c). However, when treated with NaCl, the *SOS3* and *ADH* genes were upregulated in these transgenic lines, and *SOS2*, *FRY* and *P5CS1* were downregulated (Fig. 5c). Relative to the corresponding transgenic lines without NaCl supplementation, *SOS3*, *ADH* and *P5CS1* were upregulated and the other genes were downregulated with NaCl treatments (Fig. 5c). Interestingly, in the *35S::GmPHO1;H6* transgenic lines, changes in the expression of the salt pathway marker genes were observed under both normal and salt stress conditions (Fig. 5d); however, no changes in salt tolerance were detected in the transgenic plants. Compared to the changes in gene expression patterns in the *35S::GmPHO1;H6* transgenic lines (Fig. 5d), the upregulation of the *ADH* gene in both *35S::GmPHO1;H9* and *35S::GmPHO1;H8* (Fig. 5b, c) might play an essential role in enhancing salt tolerance in the two transgenic analyses. Nonetheless, changes in the expression of the marker genes in the salt-tolerance pathways in overexpressing *GmPHO1;H6* are suggestive of the inherent nature of *PHO1* genes in salt response.

#### GmPHO1 is involved in the responses to Pi-starvation in transgenic Arabidopsis

**Fig. 6:**
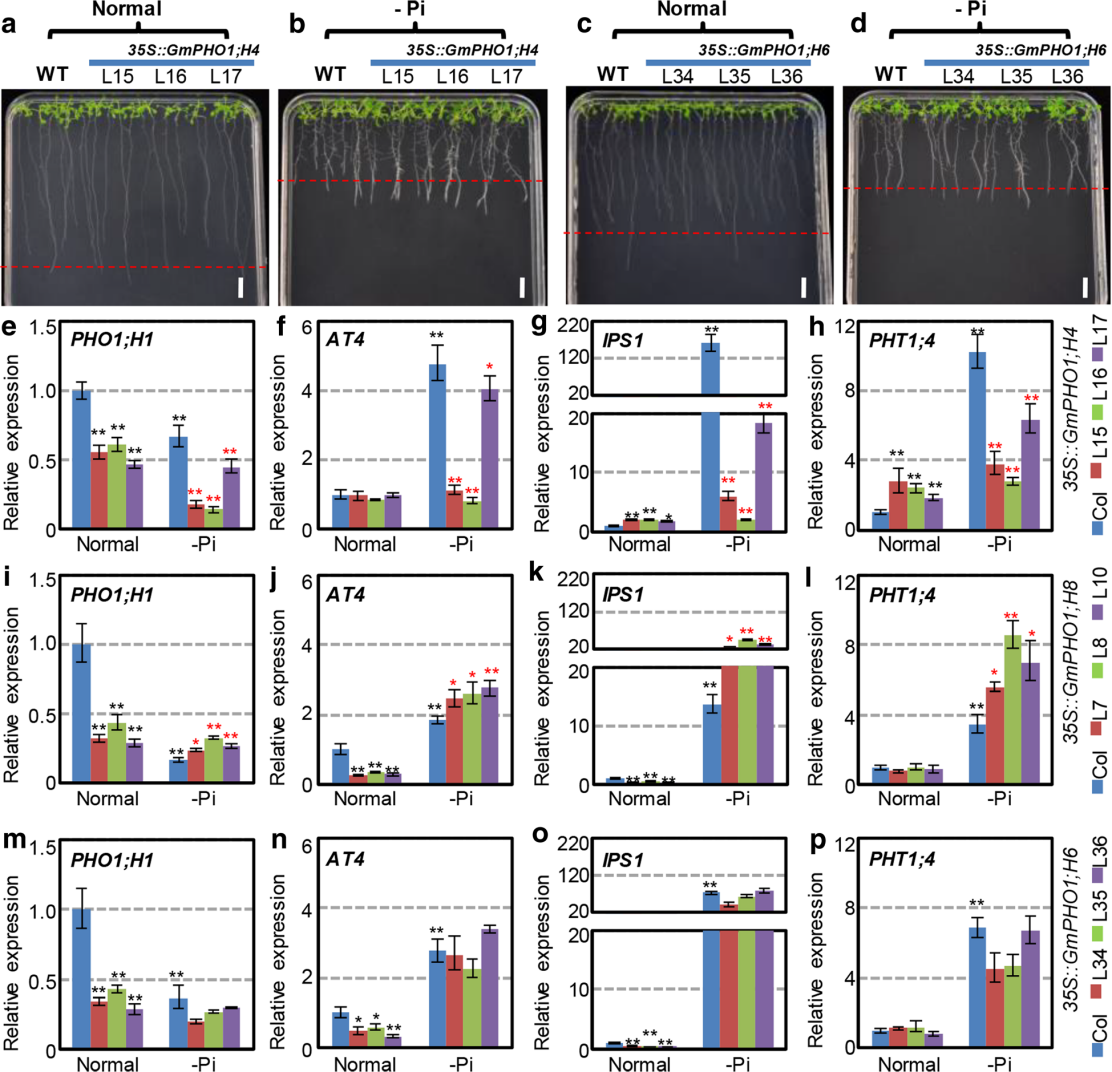
Altered sensitivity to Pi-deficiency in *GmPHO1* transgenic *Arabidopsis*. **a, b** *35S::GmPHO1;H4* transgenic lines were grown on normal (1.25 mM Pi) (**a**) and -Pi (0 mM Pi) medium (**b**) for 2 weeks. **c**, **d** *35S::GmPHO1;H6* transgenic lines were grown on normal (1.25 mM Pi) (**c**) and -Pi (0 mM Pi) medium (**d**) for 2 weeks. The red dashed line indicates the root length of the WT. Bar = 1 cm. **e**-**p** Expression of marker genes involved in the Pi pathways in transgenic *Arabidopsis.* The expression of *PHO1;H1*, *AT4*, *IPS1*, and *PHT1;4* in transgenic *Arabidopsis* plants harboring *GmPHO1;H4* (**e-h**), *GmPHO1;H8* (**i-l**), and *GmPHO1;H6* (**m-p**), respectively. Wild-type (WT) and transgenic lines grew in normal or -Pi MS medium for 2 weeks. Expression of each gene in the WT (blue column) under normal conditions was set as 1. The black stars (* or **) indicates the difference significance relative to the gene expression in WT under normal conditions, whereas the red ones indicate a significant difference relative to the gene expression in the WT under Pi-deficiency. The * means statistically significant difference at the *P* < 0.05 level, and ** indicates statistically significant difference at the *P* < 0.01 level

We further investigated the responses of *GmPHO1* transgenic plants under Pi-deficiency. The growth of primary and lateral roots, which are considered as diagnostic traits of plants in response to phosphate starvation, were shown in the presence of *35S::GmPHO1;H4* and *35S::GmPHO1;H6* (Fig. 6a-d). In response to Pi-deficiency, not only WT *Arabidopsis* showed a reduction of primary root length and an increase of lateral root number, but also all transgenic *Arabidopsis* lines did (Additional file 1: Figure S7). However, we found that the transgenic plants harboring *GmPHO1;H4* and *GmPHO1;H8* showed different responses to phosphate starvation compared to the WT, whereas the transgenic *Arabidopsis* lines harboring other *GmPHO1* genes, including *35S::GmPHO1;H6* plants, did not exhibit alterations in the sensitivity to Pi-deficiency (Fig. 6a-d, Additional file 1: Figure S7). Overexpression *GmPHO1;H4* in *Arabidopsis* was associated with longer primary roots and a higher number lateral roots than the WT (Fig. 6a, b, Additional file 1: Figure S7), thereby enhancing tolerance to Pi-starvation, whereas the *GmPHO1;H8* transgenic lines displayed significantly shorter primary roots only under Pi-deficiency (Additional file 1: Figure S7), hence increasing the sensitivity of root to insufficient Pi.

#### GmPHO1 affects the Pi signaling pathway in transgenic Arabidopsis

*IPS1*, *AT4*, *PHT1;4*, and *PHO1;H1* are upregulated in *Arabidopsis* during Pi-deficiency [4–6] and are considered to be marker genes in the Pi signal pathway. To further evaluate the possible roles of the *GmPHO1* genes in the Pi pathway, we investigated the expression levels of these marker genes in transgenic *Arabidopsis* lines. The *35S::GmPHO1;H8*, *35S::GmPHO1;H4*, and *35S::GmPHO1;H6* plants were evaluated and compared to the WT. Under normal conditions, *IPS1* and *PHT1;4* were upregulated in the *35S::GmPHO1;H4* transgenic lines, whereas *PHO;H1* was downregulated, and *AT4* expression did not change (Fig. 6e-h). *PHT1;4* expression did not change, whereas *PHO;H1, IPS1*, and *AT4* were downregulated in the *35S::GmPHO1;H8* transgenic lines compared to the WT (Fig. 6i-l). The variations in the expression of these marker genes in the *35S::GmPHO1;H6* plants was similar to that in the *35S::GmPHO1;H8* plants (Fig. 6m-p)*.* These results indicate that overexpression of *GmPHO1* could affect the expression of genes that are involved in the Pi pathway.

However, under Pi-starvation conditions, we found that *IPS1*, *AT4*, and *PHT1;4* were indeed upregulated by Pi-deficiency in WT *Arabidopsis*, whereas *PHO1;H1* was significantly downregulated (comparisons between blue columns under Pi normal and deficiency conditions in Fig. 6e-p)*.* Moreover, the expression levels of these marker genes in the *35S::GmPHO1;H4* transgenic lines decreased compared to the WT, indicating their insensitivity to Pi-deficiency (Fig. 6e-h), whereas these were all upregulated in the *35S::GmPHO1;H8* transgenic lines, showing hypersensitivity to Pi-deficiency (Fig. 6i-l). However, changes in the expression of these marker genes in the *35S::GmPHO1;H6* transgenic plants in response to Pi-starvation were similar to those of the WT (Fig. 6m-p). Therefore, in the *GmPHO1* family, *GmPHO1;H4* or *GmPHO1;H8* might participate in the Pi pathway by affecting the expression of genes that are homologous to *Arabidopsis PHO;H1*, *IPS1*, *AT4*, and *PHT1;4*.

### Responses of SN14 and ZYD6 to salinity stresses and Pi-starvation

**Fig. 7:**
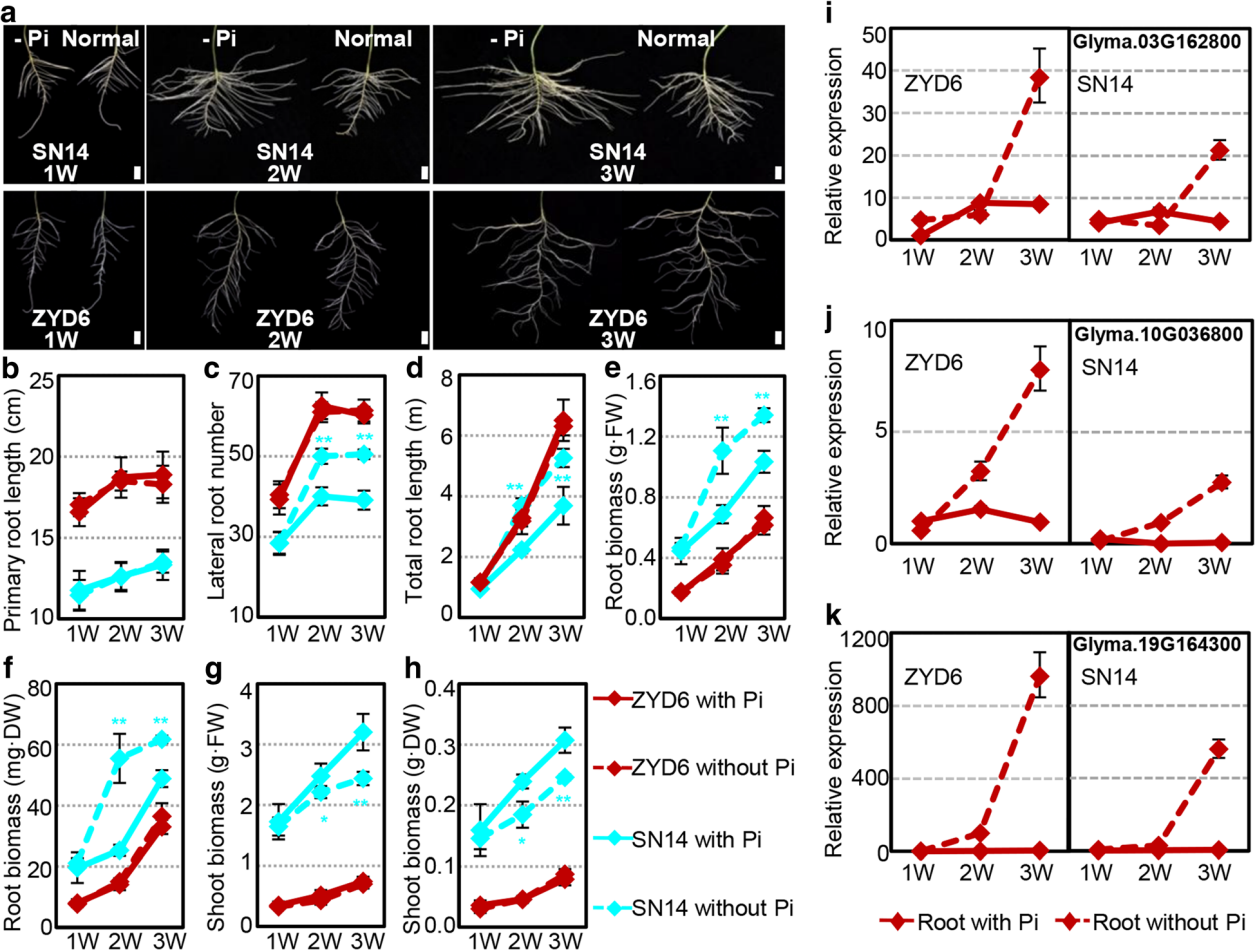
Phenotypic variations of ZYD6 and SN14 under Pi-deficiency conditions. **a** The root growth of soybeans on normal (1.25 mM Pi) or Pi-deficiency (−Pi, 0 mM Pi) Hoagland medium at the indicated time. W, week. Bar = 2 cm. **b**-**h** Quantification of phenotypic variations. **b** Primary root length. **c** Lateral root number. **d** Total root length. **e** Root biomass in terms of flesh weight. **f** Root biomass in terms of dry weight. **g** Shoot biomass in terms of flesh weight. **h** Shoot biomass in terms of dry weight. **i**-**k** The expression of the three closest homologs of *Arabidopsis PHT1;4* in soybeans under Pi-deficient conditions. Expression of each gene in the normal conditions at 1 week (W) in roots of ZYD6 was set to 1. ZYD6 and SN14 respectively represent the wild and cultivated soybean. The * means significance at the *P* < 0.05 level, and the ** represent the significance at the *P* < 0.01 level

Class II *GmPHO1* genes apparently could affect the response of transgenic *Arabidopsis* plants to stresses. Whether do these genes contribute to the divergence of soybeans in response to stresses? The native expression of *PHO1* genes in response to salinity and low-Pi stresses was diverse in the roots of SN14 and ZYD6 (Additional file 2: Table S5). However, if the expression variation pattern was compared, we found that most *PHO1* genes, particularly Class II genes, responded similarly to salinity stresses, and half of these genes had sharp contrasts in response to Pi-starvation (Additional file 2: Table S6), hinting the differential response capacities to stresses between SN14 and ZYD6. We therefore evaluated the responses of SN14 and ZYD6 to salinity stress and Pi-starvation. Using various NaCl concentrations to evaluate soybean salinity tolerance [46, 47] in SN14 and ZYD6, soybean seedlings of both accessions showed similar phenotypic variations (Additional file 1: Figure S8), indicating that the two accessions have no significant differences in salinity tolerance. However, when we treated these soybeans in low-Pi conditions, differential responses, particularly in relation to root development, were observed between SN14 and ZYD6 (Fig. 7, Additional file 1: Figure S9). We found that the primary root length of ZYD6 was longer than that of SN14 during the three-week development after germination, whereas no distinct changes in response to Pi-deficiency were observed (Fig. 7a, b). However, the development of lateral roots in SN14 was apparently promoted by Pi-deficiency, but not in ZYD6 (Fig. 7a, c). The increase in the number of lateral roots in SN14 in response to Pi-starvation was accompanied by an increase both in total root length and biomass (Fig. 7a, c-f). However, Pi-deficiency induced a significant reduction in SN14 aboveground biomass (Fig. 7g, h), but not in ZYD6, indicating that wild soybean ZYD6 is insensitive to Pi-starvation.

Based on the results of transgenic *Arabidopsis* analysis, we then checked the expression of soybean genes that were homologous to the *Arabidopsis* Pi pathway marker genes. Searching in the NCBI and Phytozome databases did not identify any homologs of *Arabidopsis IPS1* and *AT4* in soybean, whereas soybean *PHT1;4*-like genes were found. Phylogenetic reconstruction of the *PHT1;4* genes depicted the putative orthology of the soybean genes (Glyma.03G162800, Glyma.10G036800, and Glyma.19G164300) and *Arabidopsis PHT1;4* (Additional file 1: Figure S10). The total RNAs from the roots were subjected to qRT-PCR analysis, which revealed that soybean *PHT1;4*-like genes showed similar expression profiles during root development in both ZYD6 and SN14, but had different expression levels in roots. In particular, Glyma.19G164300 showed trace levels of expression under normal conditions (Fig. 7i-k). Moreover, these were all upregulated in ZYD6 and SN14 under Pi-deficiency (Fig. 7i-k). Nevertheless, the increase in the level of gene expression in response to Pi-starvation in ZYD6 was significantly higher than that in SN14.

These results suggest the differential response capacities of wild and cultivated soybeans to salinity and Pi-starvation at both phenotypic and molecular levels, which was linked with the *PHO1* gene family since the salt and Pi signaling pathways could be affected by the expression of some *GmPHO1* genes.

## Discussion

**Fig. 8:**
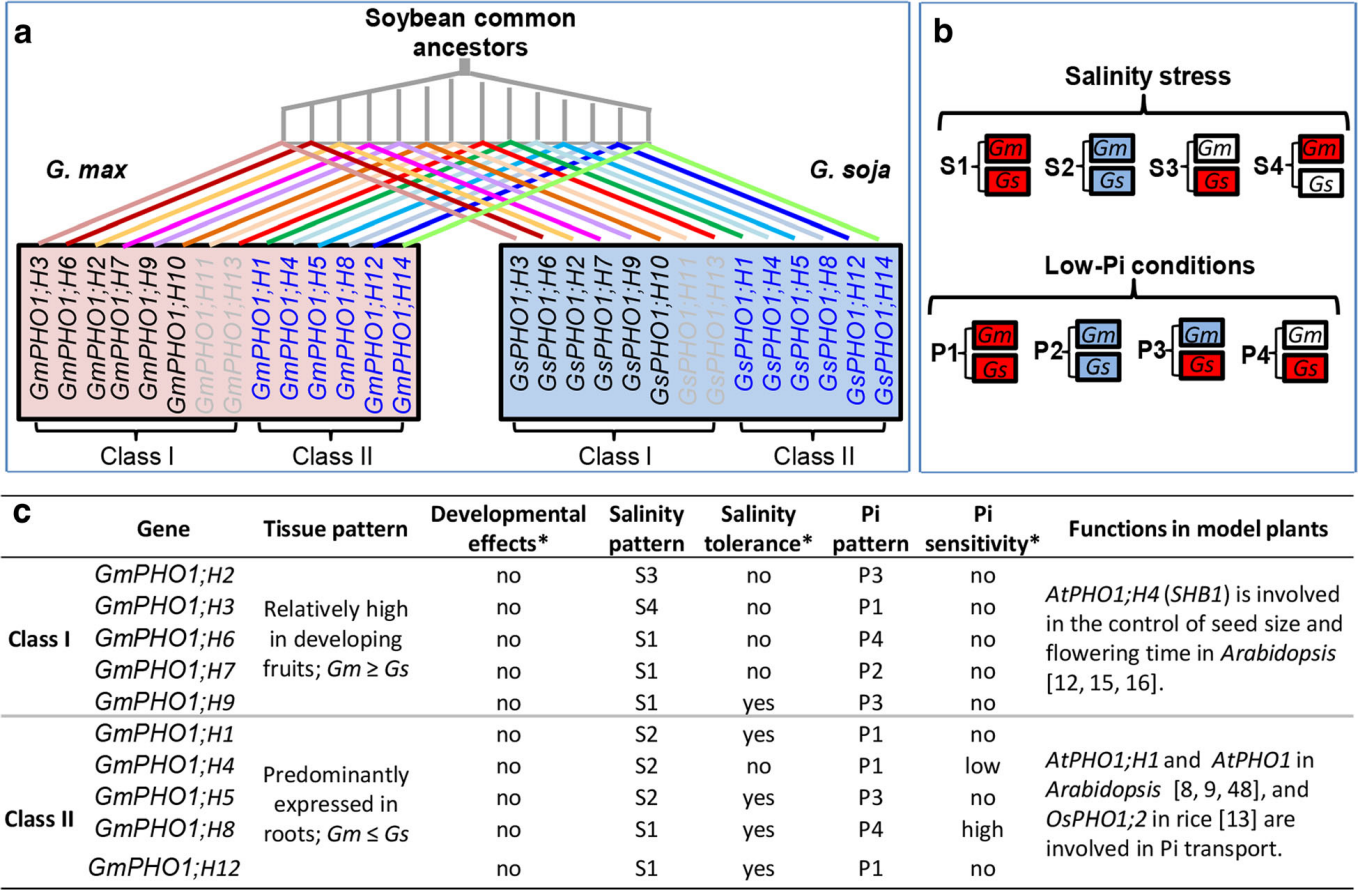
Evolution of *PHO1-like* genes during the speciation of soybeans. **a** Molecular evolution of soybean *PHO1* genes. The sequence evolution of the soybean *PHO1* gene family was conceptualized referred by the phylogenetic trees of this gene family (this work; [45]). **b**, **c** Expressional diversification of soybean *PHO1* genes and functional diversification. The transcript sequence and expression of Class I genes in gray (in **a**) were not detected, thereby suggesting pseudogenization. The expression pattern in response to the indicated stresses is summarized in roots of SN14 and ZYD6 (**b**), whereas the tissue-specific expression patterns of this gene family were inferred from the data from all accessions of *G. soja* and *G. max* that were used in this work (**c**). The response of each gene to the stresses. The box in red, blue, and white respectively indicate upregulated, downregulated, and unchanged expression in response to the indicated stresses. Overall, four patterns of gene expression variation were defined under salinity stresses (S1-S4), and low-Pi conditions (P1-P4) among wild and cultivated soybeans (for details, see Additional file 2: Table S6). *Gm*, *G. max*; *Gs*, *G. soja*. The function of soybean *PHO1* genes was inferred from transgenic *Arabidopsis* indicated by a star (*). The *PHO1* functions from model plants *Arabidopsis thaliana* and rice were included to endorse the diverse roles and functional divergence of Classes I and II in the *PHO1* gene family

The *PHO1* gene family in *Arabidopsis* participates in Pi transfer, stress responses, and regulation of seed development [8, 16, 17]. We previously investigated the phylogeny and expression of *GmPHO1* genes, suggesting potential functional divergence of its paralogs [45]. The present study investigated the divergence of *Gs-GmPHO1* orthologous gene pairs in terms of sequences and expression patterns under different conditions, evaluated their roles in determining response capacity to salinity and Pi-starvation between soybean accessions and performed transgenic *Arabidopsis* analyses (Fig. 8) to provide further insights into the functional diversification and adaptive roles of the *PHO1* gene family during soybean evolution.

### Soybean *PHO1* genes are involved in salinity or phosphate pathways

Analysis of the expression and the divergence pattern of soybean *PHO1* genes have suggested the role of this gene family in plant and seed development. However, overexpression of soybean *PHO1* genes in transgenic *Arabidopsis* did not reveal any variations in seed germination, flowering time, seedling morphology, plant height, and seed size. These findings were discordant from those of *Arabidopsis PHO1* genes [15, 16], thereby suggesting that the *PHO1* genes might have functionally diverged in plant development. The expression of soybean genes in response to salinity and phosphate deficiency suggests that these may play roles in these processes, and transgenic plants harboring specific soybean *PHO1* genes have revealed alterations in tolerance to either salinity stress or Pi-deficiency.

Overexpressing the *GmPHO1* genes in transgenic *Arabidopsis* plants has facilitated in the identification of five members of this gene family (*GmPHO1;H1*, *GmPHO1;H5*, *GmPHO1;H8*, *GmPHO1;H9*, and *GmPHO1;H12*) that improve salt tolerance in *Arabidopsis*. The germination rate, the green rate, and growth under salt stresses either was not affected or even much better than the WT. We further investigated the expression of the *SOS2*, *SOS3*, *ADH*, *P5CS1*, and *FRY1* genes, which are involved in salt tolerance pathways [21–26]. *GmPHO1* genes apparently have the capability to affect the expression of these genes using various mechanisms, thus influencing salt tolerance in the transgenic *Arabidopsis* plants. The molecular interaction between the *GmPHO1* family and salt tolerance pathways requires further investigation. However, not all genes confer salt tolerance to the transgenic *Arabidopsis* plants, possibly due to variations in the molecular interactions during evolution. Nevertheless, the phenotype could likely depend on how the transgenes affect *ADH* expression. The increase in *ADH* expression seems to be essential to conferring strong salt tolerance to the transgenic *Arabidopsis* plants.

Overexpressing *GmPHO1* in transgenic *Arabidopsis* plants demonstrated that *GmPHO1;H4* and *GmPHO1;H8* could also affect tolerance to Pi-deficiency. These observations agree with the findings in various plants. In *Arabidopsis*, *PHO1* and *PHO1;H1* are involved in phosphate transfer from the roots to the shoots of the xylem [6, 8, 48]. The *PHO1* genes have also been shown to participate in Pi transfer in rice and potato [13, 49]. Various genes such as *IPS1*, *AT4*, and *PHT1;4* are considered as marker genes in *Arabidopsis* in response to Pi-starvation [4, 5], and *pho1* shows all the hallmarks associated with Pi-deficiency, including poor shoot growth and overexpression of numerous Pi deficiency-responsive genes [2, 11]. *PHO1* expression in response to low Pi is regulated by the transcription factor WRKY6, which is degraded by a ubiquitin E3 ligase in *Arabidopsis* [9, 48]. This regulatory mechanism requires further investigation in soybean. However, we found that the expression of the abovementioned marker genes of the Pi signaling pathways in *GmPHO1* transgenic plants were differentially affected, which in turn supports notion on the diversity of tolerance to Pi-starvation. Compared to the WT under low-Pi conditions, the expression of all the four marker genes of the Pi signal pathways increased in the *35S::GmPHO1;H8 Arabidopsis* plants, indicating their hypersensitivity to low-Pi conditions, which was accompanied by shortening of the roots and suggesting that root development is inhibited in *35S::GmPHO1;H8* transgenic *Arabidopsis* plants with Pi-starvation. However, the opposite was observed in *35S::GmPHO1;H4* plants. The expression patterns of these marker genes in *35S::GmPHO1;H6* plants were similar to those of *35S::GmPHO1;H8* plants under normal conditions. However, relative to the WT, no changes in the expression of these marker genes were observed in the *35S::GmPHO1;H6* plants when challenged with low-Pi conditions, and no phenotypic variations were detected. These results indicate the complexity of the regulatory mechanism underlying the response to low-Pi levels. Nevertheless, the homologs of *Arabidopsis PHT1;4* in soybeans were differentially expressed between SN14 and ZYD6, thus further corroborating the distinct responses to low-Pi treatment between the two soybean species/accessions.

Transgenic analysis also showed that some transgenic plants harboring a few *GmPHO1* genes such as *GmPHO1;H8* showed strong tolerance to salinity stress and elevated sensitivity to Pi-deficiency, suggesting a possible interaction between the two abiotic signaling pathways. However, the molecular details of this possible crosstalk and how *GmPHO1* genes affect plant fitness (including yields, biomass accumulation etc.) in transgenic *Arabidopsis* plants require further investigations. Nevertheless, these results indicate that soybean *PHO1* genes are involved in either salinity or Pi pathways, which are conserved among plant species [8, 9, 13, 48], further suggesting the functional divergence of the soybean PHO1 paralogs in response to abiotic stresses.

### The evolutionary implication of soybean *PHO1* genes

Cultivated soybeans were domesticated from wild soybeans at least 5000 years ago [28, 29]. Variations in either coding sequence or expression of the domesticated genes are the main causative forces affecting phenotypes during plant domestication [50–55]. Differential expression of *PHO1* genes in developing fruits was detected between the wild and cultivated soybeans; however, this was not functionally linked to any fruit-related domesticated trait. Wild soybean is endowed with some excellent agronomic traits such as stress tolerance due to the wide geographic distributions and rich genetic variations [43, 44]. However, unlike seed size, the adaptive variation does not seem to be a domestication trait, but a result of diverse challenges in the environments. Elite allelic variations in certain genes involved in the related pathways in either wild or cultivated accessions could confer tolerance to certain stresses. We observed that cultivated soybean SN14 and wild soybean ZYD6 showed distinct sensitivities to low Pi, although no obvious difference in tolerance to salinity was detected. A comparative study of the *PHO1* genes between the two accessions would provide insights to the observed differences. This gene family evolved from the soybean common ancestors and showed overall similar variation patterns during soybean evolution (Fig. 8a-c). The soybean *PHO1* genes from both *G. max* and *G. soja* were divided into two classes, namely, Class I and Class II. Fourteen members of the *PHO1* family were predicted in soybean [45]; however, in the present study, we confirmed that 12 members are expressed and thus, pseudogenization may have occurred in Class I of both *G. max* and *G. soja*. The actively expressed genes in Class I were basically present in all evaluated organs, whereas the genes from Class II predominantly accumulated in the roots, thereby suggesting functional divergence of the two classes. The restricted expression of Class II *PHO1* genes in the roots could limit their role in root development and adaptation. This notion is further supported by the observations of gene expression variations in soybean roots of these *PHO1* genes in response to salinity stress and Pi-starvation.

Most *PHO1* genes showed similar expression tendency in roots between SN14 and ZYD6 (Fig. 8b, c), thus conferring a similar salt tolerance between the two accessions, while the interactions among *PHO1* genes, particularly two genes (*GmPHO1;H4* and *GmPHO1;H8*) from Class II seemed to display an essential role in conferring soybean tolerance to low Pi. Moreover, transgenic *Arabidopsis* analysis further substantiated these notions and revealed that manipulating the *PHO1* family genes could alter the plant tolerance to salinity stresses or low-Pi treatments (Fig. 8c). These observations suggested that *PHO1* gene family is involved in determining the tolerance to salinity and low-Pi stresses. Particularly, as confirmed by transgenic *Arabidopsis* analysis, *GmPHO1;H4* and *GmPHO1;H8* were potentially functional members conferring soybean sensitivity to low Pi (Fig. 8c), which might be fulfilled by affecting the expression of *PHT1;4* genes, since the *Arabidopsis PHT1;4* gene, which plays essential role in Pi acquisition [5], was significantly affected in the transgenic *Arabidopsis* plants. Moreover, the expression of *PHT1;4* genes in response to low Pi were significantly higher in ZYD6 than that in SN14, correlating to differential response capacities to Pi-deficiency between the two accessions that ZYD6 was insensitive than SN14. The relationship between *PHO1* and *PHT1;4* genes in soybean needs further investigations, however, our results suggest the functions and the divergence of the soybean *PHO1* gene family in relation to plant adaptation during evolution. Nevertheless, not all *GmPHO1* members altered the tolerance to salinity and low Pi in transgenic *Arabidopsis* plants (Fig. 8c), suggesting the functional divergence between soybean paralogs, which might be mainly due to variations in the coding sequences after gene duplications.

The *PHO1* orthologous genes in *G. max* and *G. soja* had arisen from common soybean ancestors (Fig. 8a). We found that the 12 active *PHO1* orthologous gene pairs between wild and cultivated soybeans show limited sequence divergence, and all the detected variations were predicated to be neutral, suggesting conserved functions of *Gm-GsPHO1* orthologous gene pairs between the wild and cultivated soybeans. However, we have also revealed that the *Gm-GsPHO1* orthologous gene pairs display a diverse tissue-specific expression patterns during fruit developments and gene expression patterns in response to various abiotic stresses, including salinity and Pi-deficiency, suggesting that domestication affects their expression. Interestingly, the level of *PHO1* gene expression in developing fruits of *G. max* was overall higher than that of *G. soja*, whereas the *PHO1* expression in roots of *G. soja* was generally higher than that in *G. max*, indicating the functional divergence of these genes during soybean domestication. In particular, the expression of Class I *PHO1* genes has been broadly extended to various tissues and it showed differential expression between wild and cultivated soybeans. This differential expression patterns may be involved in multiple developmental roles and be possibly associated with phenotypic divergence during soybean domestication because Class I gene *AtPHO1;H4* (*SHB1*) plays developmental roles in seed size and flowering time in *Arabidopsis* [12, 15, 16]. However, transgenic *Arabidopsis* analysis of soybean *PHO1* genes did not support this notion (Fig. 8c). The roles of soybean genes in response to salinity and low-Pi stresses were well evidenced in transgenic *Arabidopsis* studies, which is in line with the observation that the expression of soybean *PHO1* genes diversely responded to stresses, and the overall four patterns of gene expression occurred in response to salinity (S1-S4) or low Pi (P1-P4) conditions in soybeans, where some *Gm*-*GsPHO1* orthologous pairs had similar response patterns with different extent under either salinity stress or Pi-deficiency. However, others had quite different expression variations both in expression trend and strength, even a contrary tendency (Fig. 8b). *PHO1* genes differentially responded to salinity and low-Pi stresses in the two soybean accessions; however, only differential responses to low Pi were observed, suggesting that *PHO1* genes are primarily involved in the Pi pathways, whereas the role of these genes in salinity stresses, which was reflected in transgenic *Arabidopsis*, could be masked by variations in other soybean genes. The molecular basis underlying the responses to salinity and low Pi in soybean might be highly complex. However, the divergence of expression and its response to stresses of *PHO1* gene family in the roots of SN14 and ZYD6, if this could be generalized between *G. max* and *G. soja*, could play a role in differentiating the capability of stress signal perception and transduction, hence tolerance to certain stresses between the two species during the evolution.

### Functional diversification of the plant *PHO1* genes

The *PHO1* gene family plays multiple roles during plant development and evolution. *Arabidopsis SHB1* (*PHO1;H4*), which is involved in flowering time and seed size control [12, 15, 16], belongs to Class I. Transgenic *Arabidopsis* analysis did not show any evidence supporting such a role for *PHO1*-like genes in soybean. However, such a role was not excluded because the expression of Class I genes had diverged between *G. max* and *G. soja* during fruit development. Alternatively, its role in seed size might be specific to the *Brassica* species or only in *Arabidopsis*. Class I genes show broad and diverse expression patterns, which is indicative of multiple developmental roles. The role of Class II members in low Pi is particularly conserved in various plants, including *Arabidopsis* [6, 8], rice [13], cultivated soybean [45], and potato [49], suggesting a specific role for Class II *PHO1* genes in the Pi signaling pathways. This observation to a certain extent corroborates the different responses of SN14 and ZYD6 to low-Pi treatments. We also have elucidated the role of soybean *PHO1* genes in responses to salinity stresses, and in transgenic *Arabidopsis*, most genes from Class II alter tolerance to salinity stresses. However, only one gene *GmPHO1;H9* from Class I did, further supporting functional divergence between the two classes. However, differential expression of these *PHO1* genes apparently could not confer differential salinity tolerance to SN14 and ZYD6, further reflecting the complexity of the molecular mechanism underlying plant responses to abiotic stresses. The plant *PHO1* gene family has diverse roles, which were determined by expression divergence (Fig. 8b, c), although the molecular variations underlying the expression divergence needs further investigation. The expression of all soybean genes in the whole family in response to various abiotic stresses seems to be diversely active, implying that the *PHO1*-like genes are primarily involved in the adaptive evolution of plants.

## Conclusions

Taken together, significant variations in gene expression among different tissues, particularly in developing fruits and roots, and its variations in roots in response to low-Pi and salinity stresses largely underlies the functional divergence of the orthologous gene pairs of the *PHO1* family between wild and cultivated soybeans. No direct functional evidence was observed to support their role in soybean fruit development, however, the expression variation in roots matters in determining the response capacities in different soybean accessions. The modes of functional evolution of the soybean *PHO1* genes are overall unclear, but transgenic *Arabidopsis* analyses have revealed functional divergence between members of this gene family in response to salinity and low-Pi stresses, thus endorsing that the *PHO1* genes play an adaptive role in soybean evolution. The mechanisms of the soybean *PHO1* genes in relation to various stresses, their crosstalk, and roles of adaptive evolution need further investigations in the native hosts. This study also provides promising genetic materials for crop improvement; i.e., genetically manipulating *GmPHO4*, *GmPHO8*, and *GmPHO9* could improve soybean performance under salinity stresses or low-Pi conditions.

## Methods

### Plant materials and growth conditions

The cultivated soybean Suinong14 (SN14) and the wild accession ZYD00006 (ZYD6) [56] were grown in a greenhouse of the Institute of Botany (Beijing, China) under long-day conditions (16 h light/8 h dark at 23 °C–25 °C). Roots, leaves, and stems of three-week-old seedlings were collected, and mature flowers and 1-, 3-, 5-, or 15-day post-fertilization fruits were gathered. Biological samples from four additional wild (Y1, Y2, Y3, and Y4) and cultivated (Hefeng48, Nenfeng16, Heinong35, and Dongnong53) soybeans [57] were included for tissue-specific expression profiling. The soybean seeds are available upon request for research only.

### Salinity stresses and low-Pi treatment of soybeans

For salt stresses, seeds of SN14 and ZYD6 were germinated in vermiculite in a chamber with 1/5 Hoagland solution for 2 weeks and then supplemented with 1/5 Hoagland solution with different NaCl concentrations (100 mM, 150 mM, 200 mM, and 250 mM). Soybean seedlings without salinity treatment were used as controls. The roots were harvested after treatments for 4 h for gene expression study.

For low-Pi treatments, seeds of SN14 and ZYD6 were germinated and cultivated in a chamber with 1/5 Hoagland solution with normal concentration Pi (1.25 mM) or without Pi for 3 weeks. The roots and leaves were respectively harvested after one, two, and 3 weeks of treatments for gene expression profiling.

Each treatment consisted of three replicates. For phenotypic observations, the same solution was supplemented every 2 days in salinity stresses or refreshed every week in low-Pi treatments until the observed time as indicated.

### Quantitative RT-PCR (qRT-PCR) analysis

Total RNA was extracted using SV Total RNA Isolation System (Promega, Madison, WI, USA), and subsequently treated with DNase I (Promega) to digest the residual DNA. The cDNA was synthesized with oligo (dT)_18_ primers following the instructions of the M-MLV cDNA synthesis kit (Invitrogen, Carlsbad, USA). qRT-PCR was performed on an Mx3000P QPCR system (Agilent, CA, USA) using SYBR Premix Ex Taq (TaKaRa, Dalian, China). Soybean *ACTIN* gene (Glyma18g52780) was used as internal control. Each experiment was performed using three independent biological samples, and means ± standard deviations are presented. The gene-specific primers for *PHO1* family were designed as previously described [45]. Primers for the *Arabidopsis* marker genes of the Pi signaling pathways and the salt tolerance pathway genes were respectively synthesized as described in the previous studies [3, 56]. All primer sequences designed in this work are presented in Additional file 2: Table S7.

### Transgenic *Arabidopsis* analysis

The open reading frames (ORF) of the *GmPHO1* genes were inserted into a pCAMBIA1300 vector that was driven by a cauliflower mosaic virus 35S promoter. Each construct was transformed into *Arabidopsis thaliana* as mediated by *Agrobacterium tumefaciens* strain GV3101 using the floral dipping method [58]. The transgenic plants were selected on Murashige & Skoog (MS) medium containing 35 mg/L hygromycin (Roche, Basel, Switzerland) and confirmed by RT-PCR. *Arabidopsis* plants were grown in a growth chamber under long-day conditions (16 h light/8 h darkness at 23 °C–25 °C).

Seeds of the T_3_ transgenic *Arabidopsis* plants were sterilized using 70% ethanol (v/v) for 3 min and in 15% NaClO (v/v) for 10 min. These were then washed four to five times with sterile ddH_2_O and stratified for 3 days at 4 °C before transfer to a growth chamber. The seeds were germinated on 1/2 MS medium for 1 week and then transferred to the soil for observing the phenotype of transgenic plants. Germination rates were also checked at 4 days. The number of rosette leaves at bolting was recorded for the flowering time. The height of seven-week-old seedlings was measured. The seeds were weighted using a laboratory balance (Mettler-Toledo, Zurich, Switzerland) and photographed under a stereomicroscope (Nikon, Tokyo, Japan).

### Salt treatments of transgenic *Arabidopsis* lines

For the observation of green rate, seeds were plated on 1/2 MS medium containing two concentrations of NaCl (125 mM and 175 mM) for 6 weeks. Germination rate was recorded at two, four, and six days after plating. To further observe salinity tolerance of transgenic lines, seedlings were grown on 1/2 MS medium for 1 week, and then transferred to the soil for 3 weeks. These plants were treated with 250 mM NaCl (once every 5 days). Approximately 15 days after treatment, the bleaching degree of plants and the content of chlorophyll were measured. Then, the aboveground biomass at the 30th day of treatment was also measured. One-week-old seedlings grown on 1/2 MS were transferred to a medium with 175 mM NaCl for 24 h, and the whole seedlings were harvested to investigate the expression of salt tolerance pathway genes.

### Low-Pi treatments of transgenic *Arabidopsis* plants

Seeds were germinated on 1/2 MS with low-Pi and normal-Pi medium for 2 weeks. Low (0 mM KH_2_PO_4_) and normal (1.25 mM KH_2_PO_4_) Pi medium were modified based on Murashige and Skoog medium with pH 5.7, 0.5% (w/v) sucrose, and 0.4% (w/v) phytagel. The basic medium was prepared as previously described [59].

### Determination of chlorophyll content

Aerial parts of the *Arabidopsis* plants were collected and crushed in absolute alcohol. The mixture was rapidly shaken and left to stand in the dark for 16 h. The absorbance of supernatant was recorded at wavelengths of 663 nm and 645 nm using a UV-visible spectrophotometer (ChemitoSpectrascan, UV 2600). Chlorophyll content was estimated according to Arnon (1949) [60].

### Soybean morphological analysis

After salinity stresses and low-Pi treatments, the seedlings were photographed using a camera (Nikon, Tokyo, Japan). In particular, after low-Pi treatments, root length was measured using a WinRHIZO root analysis system (WinRHIZO, Regent, Canada), and the number of roots was manually counted. The fresh and dry biomass was recorded using laboratory balance (Mettler-Toledo, Zurich, Switzerland).

### Statistical analyses

Each experiment/measurement was performed using three independent biological replicates or repeated three times unless stated otherwise. A student’s two-tailed *t*-test was used for statistical analysis, which was performed by using IBM SPSS Statistics for Windows, Version 24.0 (IBM Corp, New York, NY, USA).

### Sequencing analyses

The *PHO1*-like genes in Williams 82 was characterized in our previous work [45]. The gene-specific primers were designed to obtain the full cDNA sequences of SN14 (*GmPHO1*) and ZYD6 (*GsPHO1*). The unrooted neighbor-joining (NJ) phylogenetic tree was constructed based on amino acid sequences by using MEGA5 [61]. Sequence divergence between *Gm*-*Gs*PHO1 orthologous pairs, including the substitution and in-frame insertions and deletions, was predicted using SNAP and PROVEAN [62, 63]. Sequencing was conducted by Taihe Biotechnology Company (Beijing, China). Primers used in the present study (Additional file 2: Table S7) were commercially synthesized in Taihe Biotechnology Company.

## Additional files

Additional file 1: **Figure S1**. No transcripts of *PHO1;H11* and *PHO1;H13* were detected in soybeans. **Figure S2**. Phylogenetic analysis of the PHO1 family of soybean and *Arabidopsis*. **Figure S3**. Organ-specific expression of *PHO1* gene family in ZYD6 and SN14. **Figure S4**. Organ-specific expression of *PHO1* genes among various soybeans. **Figure S5**. Molecular verification of *GmPHO1* transgenic *Arabidopsis* plants. **Figure S6**. Salt tolerance of *GmPHO1* transgenic *Arabidopsis*. **Figure S7**. Root development in transgenic *Arabidopsis* under Pi-deficiency conditions. **Figure S8**. The phenotypes of soybean seedlings under different salt stresses. **Figure S9**. Phenotype of soybean seedlings under Pi-deficiency. **Figure S10**. The NJ tree of PHT1;4-like proteins from soybean and *Arabidopsis*. (PDF 940 kb)

Additional file 2: **Table S1.** Sequence identities of the GsPHO1 and GmPHO1 proteins. **Table S2.** Variations in the GsPHO1 and GmPHO1 proteins. **Table S3.** The phenotypes of *GmPHO1* transgenic plants in *Arabidopsis*. **Table S4.** The germination rate of transgenic plants under 1/2 MS medium with different concentrations of NaCl. **Table S5.** The mRNA accumulation of *PHO1* genes in roots of SN14 and ZYD6 under different stresses. **Table S6.** The variation pattern of *PHO1* genes in response to stresses in SN14 and ZYD6. **Table S7.** Primers used in the present work. (PDF 535 kb)

## Data Availability

All relevant supporting data can be found within the Additional files accompanying this article. Sequence data described in this article can be found in GenBank (http://www.ncbi.nlm.nih.gov) under the accessions of MH668976-MH668999.

## Abbreviations

cDNA: complementary DNA
ML: maximum-likelihood
NJ: neighbor-joining
ORF: open reading frame
*PHO1*: *PHOSPHATE1*
Pi: inorganic phosphate
qRT-PCR: quantitative RT-PCR
RT-PCR: reverse transcription-polymerase chain reaction
