# Supplementary material for: The PHOSPHATE1 genes participate in salt and Pi signaling pathways and play adaptive roles during soybean evolution
Source: BMC Plant Biol. 2019 Aug 14;19:353. doi: 10.1186/s12870-019-1959-8 (PMC6694516; doi:10.1186/s12870-019-1959-8)
Supplement: Supplementary file 1 — Figure S1. No transcripts of PHO1;H11 and PHO1;H13 were detected in soybeans. Figure S2. Phylogenetic analysis of the PHO1 family of soybean and Arabidopsis. Figure S3. Organ-specific expression of PHO1 gene family in ZYD6 and SN14. Figure S4. Organ-specific expression of PHO1 genes among various soybeans. Figure S5. Molecular verification of GmPHO1 transgenic Arabidopsis plants. Figure S6. Salt tolerance of GmPHO1 transgenic Arabidopsis. Figure S7. Root development in transgenic Arabidopsis under Pi-deficiency conditions. Figure S8. The phenotypes of soybean seedlings under different salt stresses. Figure S9. Phenotype of soybean seedlings under Pi-deficiency. Figure S10. The NJ tree of PHT1;4-like proteins from soybean and Arabidopsis. (PDF 940 kb) [file 12870_2019_1959_MOESM1_ESM.pdf]

The *PHOSPHATE1* genes participate in salt and Pi signaling pathways and play adaptive roles during soybean evolution

Yan Wang[1,#], Huihui Gao[1,2,#], Lingli He[1,2,#], Weiwei Zhu[1,2], Lixin Yan[1,2], Qingshan Chen[3], and Chaoying He[1,2,4,*]

1 State Key Laboratory of Systematic and Evolutionary Botany, Institute of Botany, Chinese Academy of Sciences, Nanxincun 20, Xiangshan, 100093 Beijing, China;
2 University of Chinese Academy of Sciences, Yuquan Road 19A, 100049 Beijing, China;
3 College of Agriculture, Northeast Agricultural University, 150030 Harbin, Heilongjiang, China;
4 The Innovative Academy of Seed Design, Chinese Academy of Sciences, Beijing 100101, China.
# These authors contributed equally to this work.
* Corresponding author: chaoying@ibcas.ac.cn; Tel: 86-10-62836085; Fax: 86-10-62590843

Additional file 1: Supplementary Figures (in one PDF file)
Figure S1 No transcripts of *PHO1;H11* and *PHO1;H13* were detected in soybeans.
Figure S2 Phylogenetic analysis of the PHO1 family of soybean and *Arabidopsis*.
Figure S3 Organ-specific expression of *PHO1* gene family in ZYD6 and SN14.
Figure S4 Organ-specific expression of *PHO1* genes among various soybeans.
Figure S5 Molecular verification of *GmPHO1* transgenic *Arabidopsis* plants.
Figure S6 Salt tolerance of *GmPHO1* transgenic *Arabidopsis*.
Figure S7 Root development in transgenic *Arabidopsis* under Pi-deficiency conditions.
Figure S8 The phenotypes of soybean seedlings under different salt stresses.
Figure S9 Phenotype of soybean seedlings under Pi-deficiency.
Figure S10 The NJ tree of PHT1;4-like proteins from soybean and *Arabidopsis*.

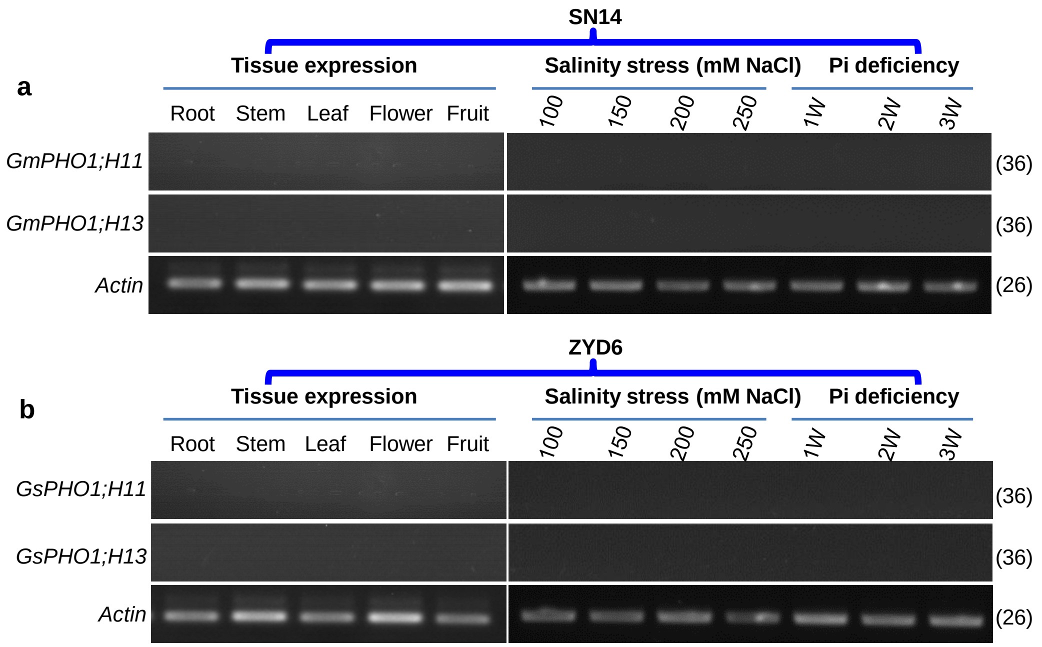

Figure S1 No transcripts of *PHO1;H11* and *PHO1;H13* were detected in soybeans. (**a**) Amplification of *PHO1;H11* and *PHO1;H13* in SN14. (**b**) Amplification of *PHO1;H11* and *PHO1;H13* in ZYD6. Total RNAs were extracted from the indicated various tissues, and roots under various stress treatments, including different concentrations of NaCl and low Pi stresses. Soybean *ACTIN* gene (Glyma18g52780) was used as a loading control. SN14, Suinong 14; ZYD6, ZYD00006; W, week. The amplification cycle of each gene is given in the corresponding parenthesis.

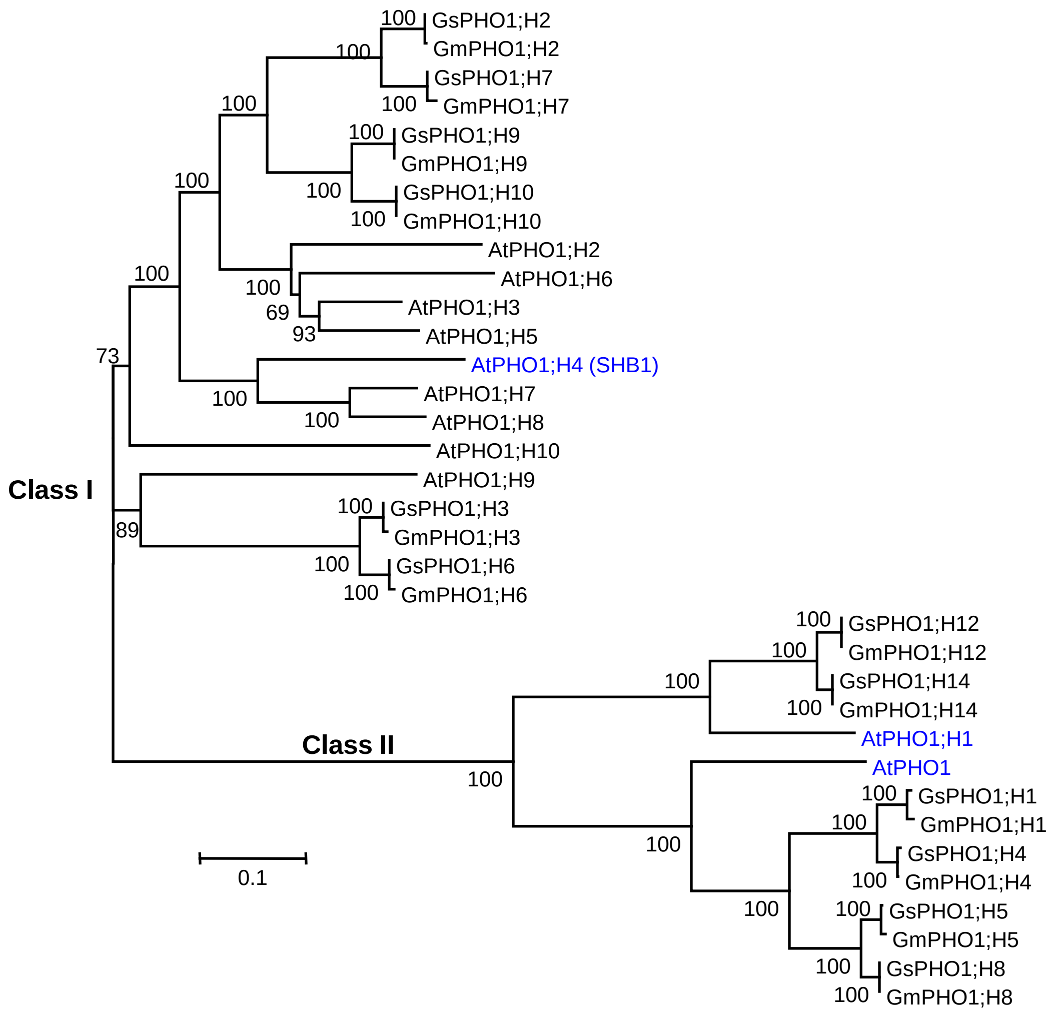

Figure S2 Phylogenetic analysis of the PHO1 family of soybean and *Arabidopsis*.
The NJ phylogenetic tree was constructed based on PHO1 protein sequences from wild soybean (ZYD6), cultivated soybean (SN14) and *Arabidopsis* respectively. Bootstrap values for 1,000 replicates are shown. Functionally well characterized *Arabidopsis* genes are highlighted in blue.

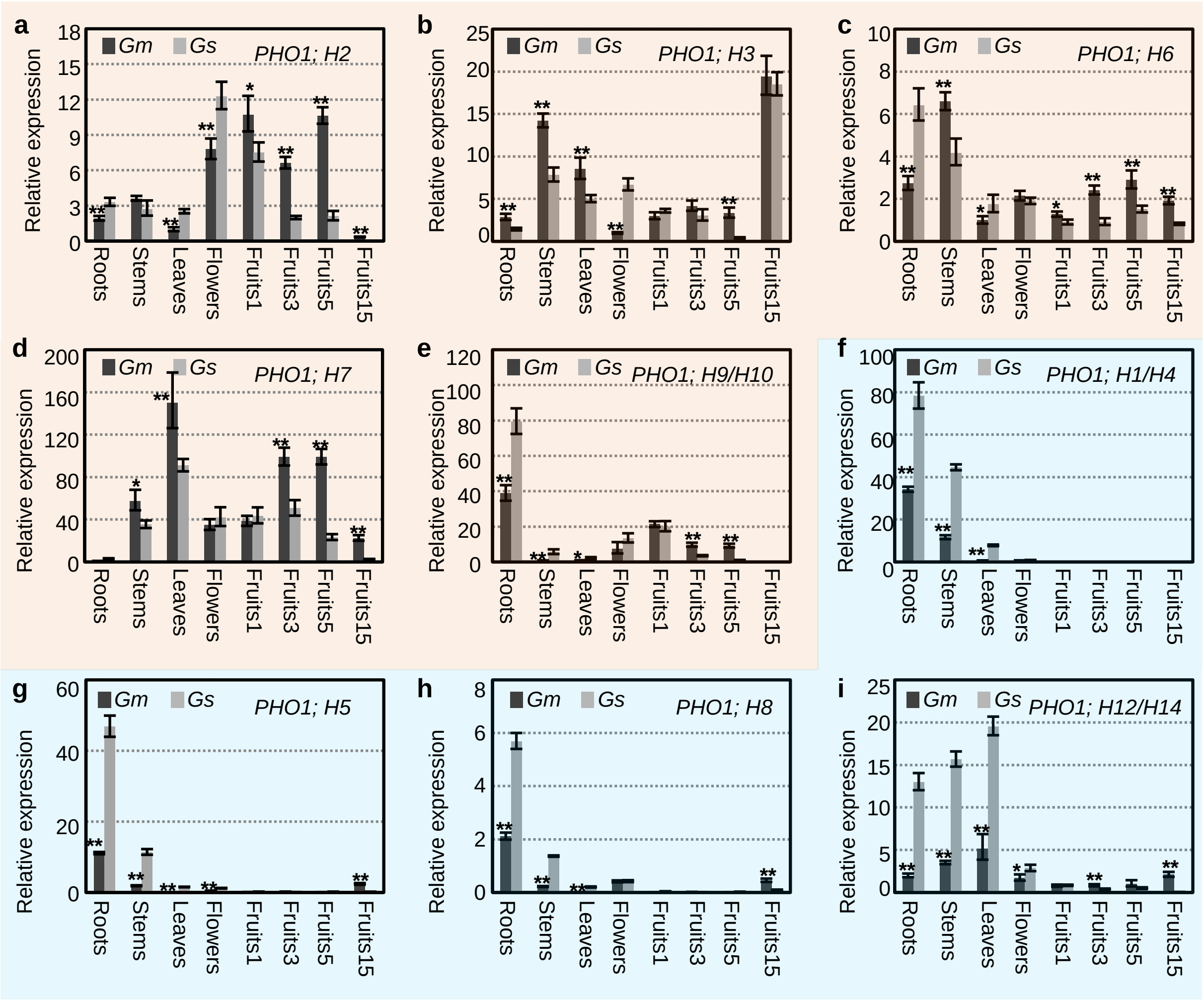

Figure S3 Organ-specific expression of *PHO1* gene family in ZYD6 and SN14.
(**a**-**e**) Organ-specific expression of *PHO1* genes in Class I. (**f**-**i**) Organ-specific expression of *PHO1* genes in Class II. Total RNAs from roots, stems, leaves, mature flowers and developing fruits (1-, 3-, 5-, and 15-day fruits after pollination) were subjected to qRT-PCR. Soybean *ACTIN* (Glyma18g52780) was used as an internal control. The experiments were performed based on three independent biological samples. Error bars: standard deviations. The * means significance at the $P < 0.05$ level, and the ** represents the significance at the $P < 0.01$ level.

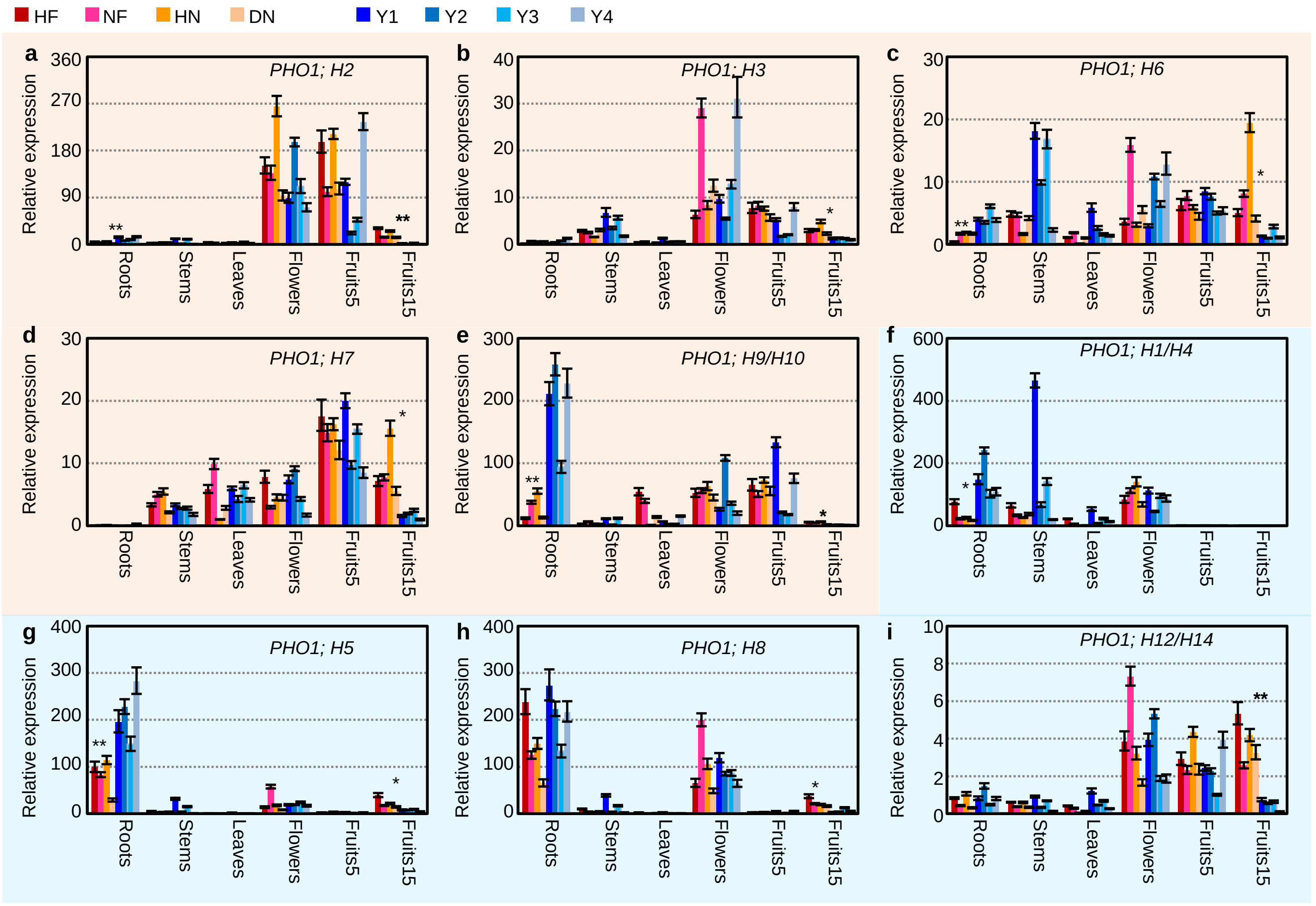

Figure S4 Organ-specific expression of *PHO1* genes among various soybeans.
(**a-e**) Organ-specific expression of the Class I *PHO1* genes. (**f-i**) Organ-specific expression of the Class II *PHO1* genes. Total RNAs from the roots, stems, leaves, mature flowers and fruits (5-, and 15-day fruits after pollination) were subjected to qRT-PCR. Four cultivated soybeans (Hefeng48, Nenfeng16, Heinong35, and Dongnong53) and four wild soybeans (Y1, Y2, Y3, and Y4) abbreviated as HF, NF, HN, DN, Y1, Y2, Y3, and Y4, respectively, were included. Differences in gene expression between cultivated and wild soybeans were evaluated using student's two-tailed *t*-test.

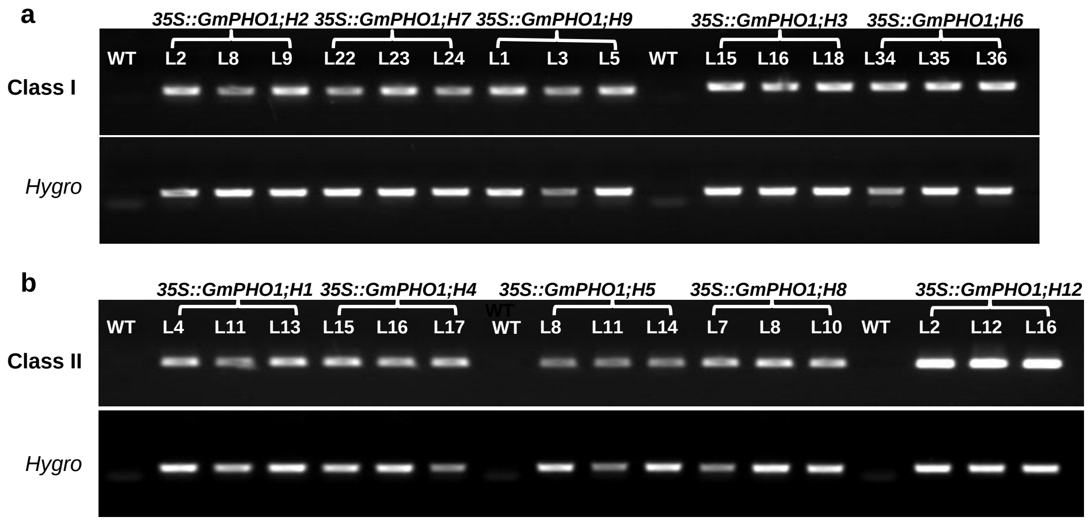

Figure S5 Molecular verification of *GmPHO1* transgenic *Arabidopsis* plants.
(**a**) Transgenic lines of Class I genes. (**b**) Transgenic lines of Class II genes. The expression of both *GmPHO1* and hygromycin resistance gene (*Hygro*) in three lines of each gene as indicated were detected using RT-PCR. WT was used as control and no *GmPHO1* genes were amplified.

a Class	Gene	Line	Seedling green rate (%) 0 mM NaCl	125 mM NaCl	175 mM NaCl
	WT		96.94±2.86	94.20±3.20	44.14±2.70
		L2	98.15±1.60	90.74±4.47	48.15±5.17
	PHO1;H2	L8	97.22±4.81	90.74±5.11	46.30±3.80
		L9	98.15±1.60	91.67±4.98	46.30±3.80
		L15	97.22±2.78	96.30±3.68	43.52±1.43
	PHO1;H3	L16	98.15±3.21	95.37±3.40	42.59±1.43
		L18	98.15±3.21	94.44±3.08	44.44±2.48
Class I		L34	99.07±1.60	96.30±3.34	42.59±3.80
	PHO1;H6	L35	97.22±4.81	95.37±3.40	46.30±2.87
		L36	98.15±1.60	96.30±3.34	44.44±2.48
		L22	97.22±2.78	93.52±3.40	40.74±5.17
	PHO1;H7	L23	97.22±2.78	94.44±3.08	41.67±2.48
		L24	99.07±1.60	93.52±2.97	42.59±3.80
		L1	96.30±4.24	96.30±3.34	72.22±2.48 **
	PHO1;H9	L3	99.07±1.60	95.37±3.40	73.15±3.80 **
		L5	99.07±1.60	96.30±3.68	73.15±1.43 **
		L4	96.30±1.60	96.30±3.34	72.22±2.48 **
	PHO1;H1	L11	99.07±1.60	95.37±3.03	73.15±3.80 **
		L13	97.22±2.78	92.59±3.80	75.93±1.43 **
		L15	97.22±4.81	95.37±2.97	40.74±2.87
	PHO1;H4	L16	96.30±1.60	96.30±3.68	38.89±2.48
		L17	97.22±2.78	93.52±3.99	44.44±4.30
		L8	95.37±4.24	96.30±3.68	63.89±2.48 **
Class II	*PHO1;H5*	L11	97.22±2.78	96.30±3.34	65.74±3.80 **
		L14	97.22±2.78	93.52±3.64	62.04±1.43 **
		L7	99.07±1.60	93.52±3.64	57.41±3.80 **
	PHO1;H8	L8	97.22±2.78	95.37±3.40	63.89±2.48 **
		L10	98.15±3.21	93.52±3.99	62.04±3.80 **
		L2	99.07±1.60	94.44±3.08	61.11±2.48 **
	PHO1;H12	L12	98.15±3.21	92.59±4.59	58.33±4.97 *
		L16	97.22±2.78	93.52±3.80	62.96±3.80 **

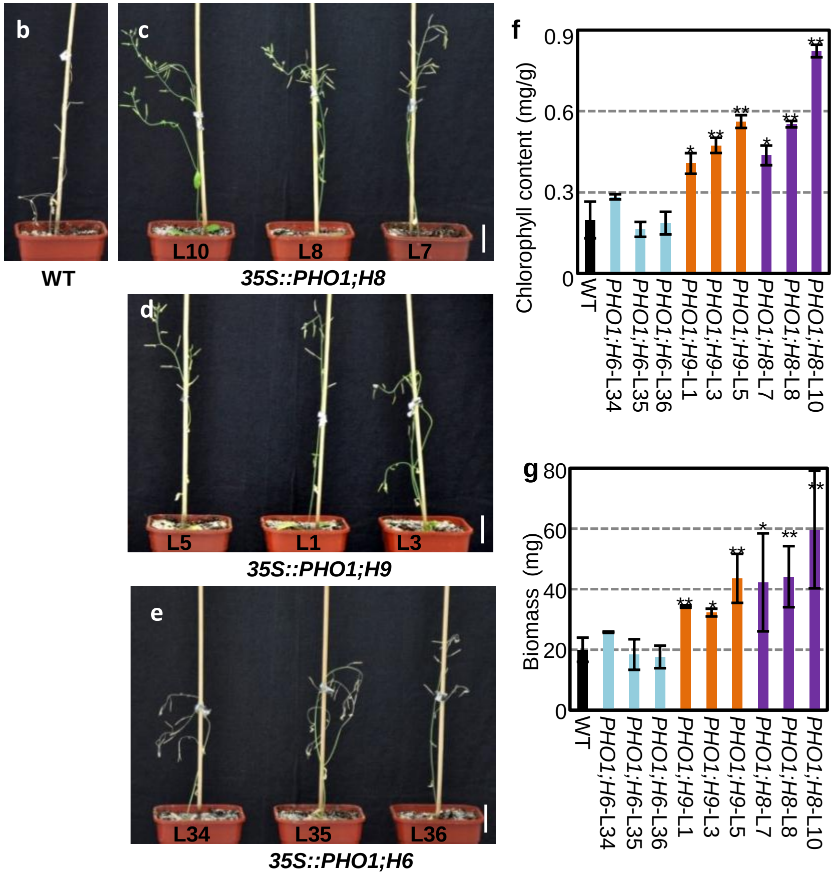

Figure S6 Salt tolerance of *GmPHO1* transgenic *Arabidopsis*.
(**a**) The seedling greening rate of *GmPHO1* transgenic plants. (**b**) WT (**c**) *35S::GmPHO1;H8* (**d**) *35S::GmPHO1;H9* (**e**) *35S::GmPHO1;H6*. WT and transgenic *Arabidopsis* lines were grown in soil supplemented with 250 mM NaCl for 15 days. Bar = 2 cm. (**f**) The chlorophyll content in transgenic *Arabidopsis* lines and WT after treatment with 250 mM NaCl in soil for two weeks. (**g**) The biomass of transgenic *Arabidopsis* lines and WT under the NaCl treatment for one month. The significance was tested in comparison with the WT. The * means significance at the $P < 0.05$ level, and the ** represents the significance at the $P < 0.01$ level.

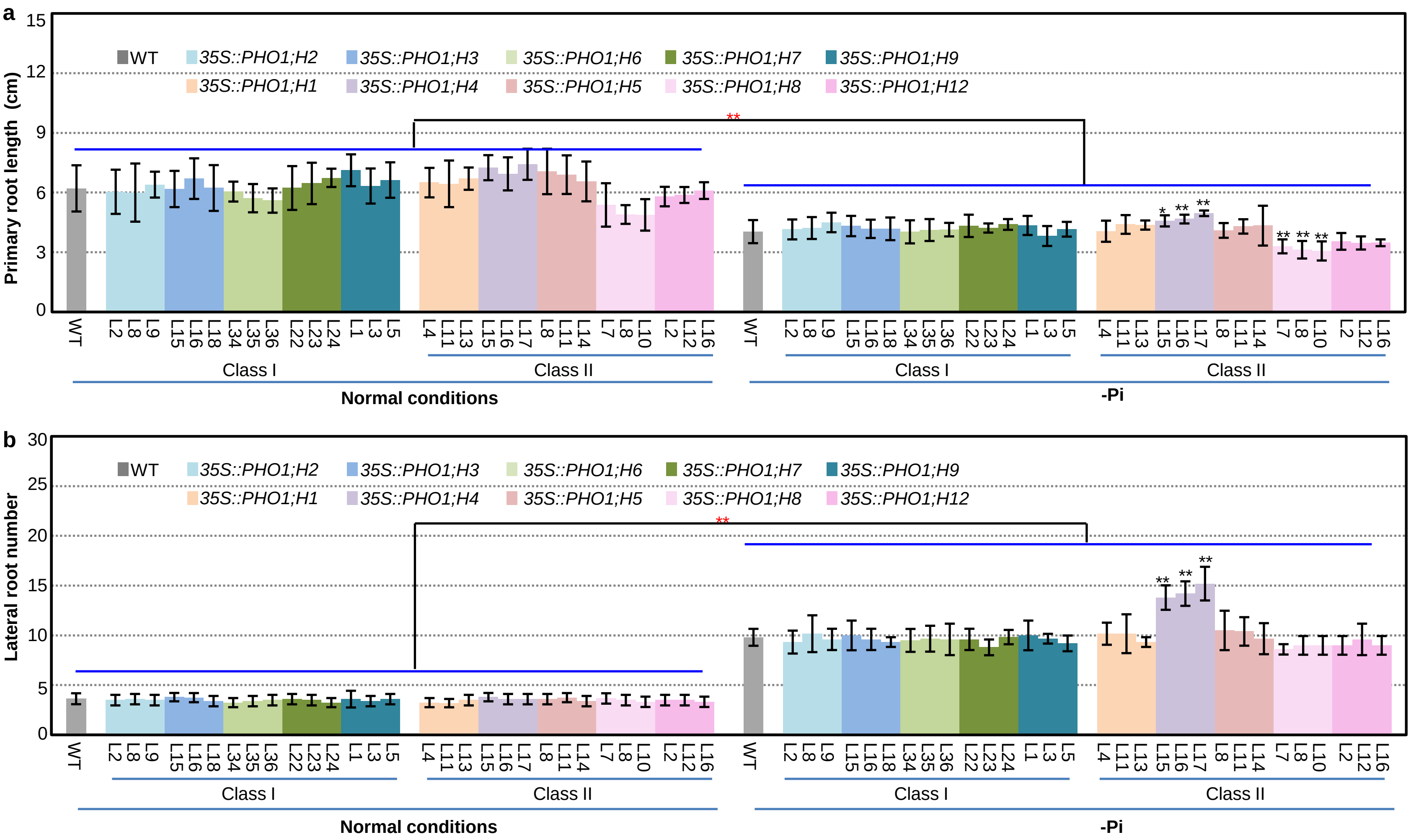

Figure S7 Root development in transgenic *Arabidopsis* under Pi-deficiency conditions.
(**a**) Primary root length. (**b**) Lateral root member. They were measured in wild-type (WT) and all *GmPHO1* transgenic *Arabidopsis* lines grown in normal (1.25 mM Pi) and -Pi (0 mM Pi) medium for two weeks. Significance analysis between the transgenic lines compared to WT under the same conditions were shown in black star. Significant differences between normal and stressed groups were indicated in red star. The * means significance at the $P < 0.05$ level, and the ** represent the significance at the $P < 0.01$ level.

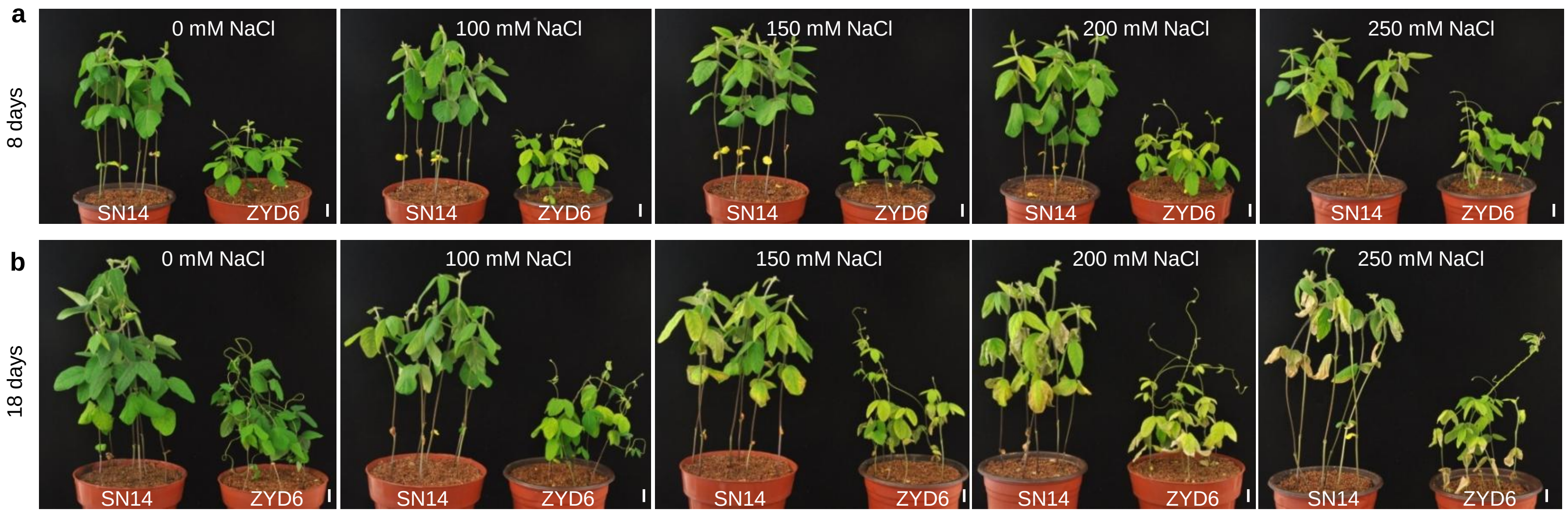

Figure S8 The phenotypes of soybean seedlings under different salt stresses.
(**a**) Phenotypes of SN14 and ZYD6 under NaCl stresses for eight days. (**b**) Phenotypes of SN14 and ZYD6 under NaCl stresses for 18 days. The two-week-old seedlings of the wild (ZYD6) and cultivated soybean (SN14) were treated under various NaCl concentrations as indicated. Bar = 2 cm.

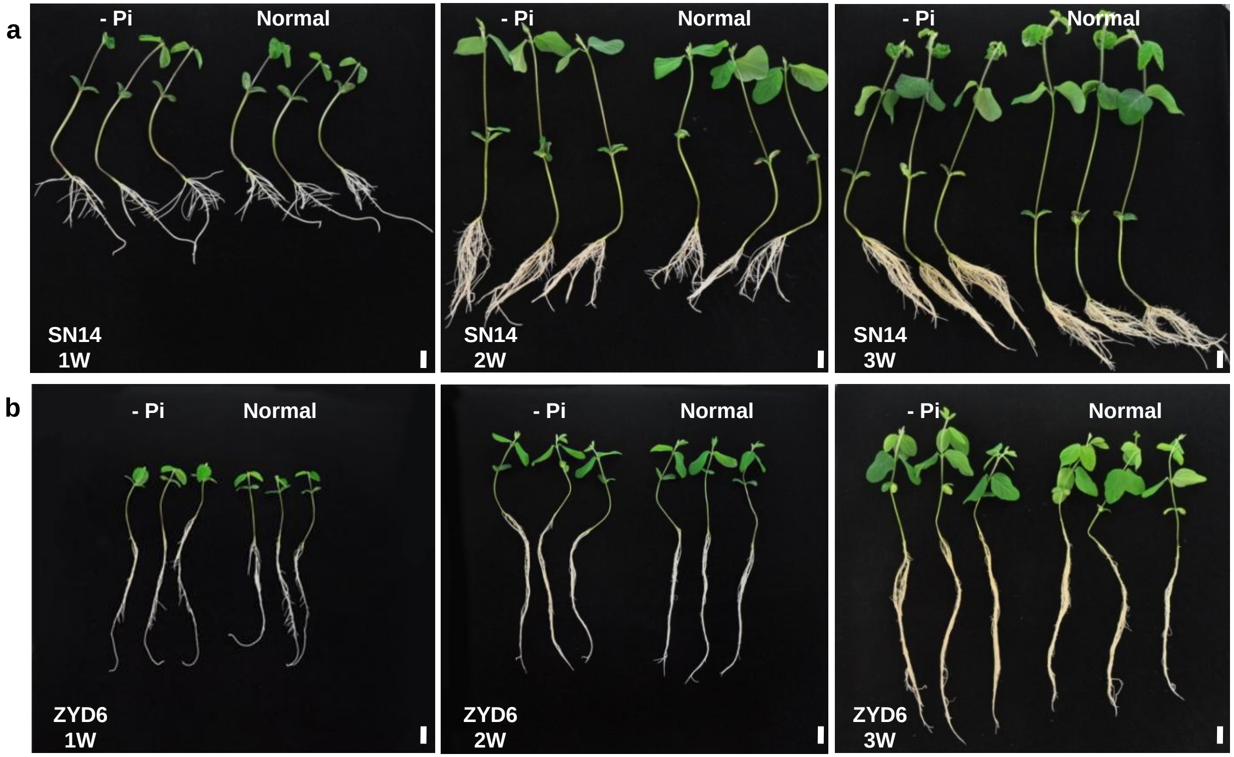

Figure S9 Phenotype of soybean seedlings under Pi-deficiency.
(**a**) Phenotype of SN14. (**b**) Phenotype of ZYD6. Wild (ZYD6) and cultivated soybean (SN14) were grown on normal (1.25 mM Pi) or Pi deficiency (-Pi, 0 mM Pi) Hoagland medium as indicated time. W, week. Bar = 2 cm.

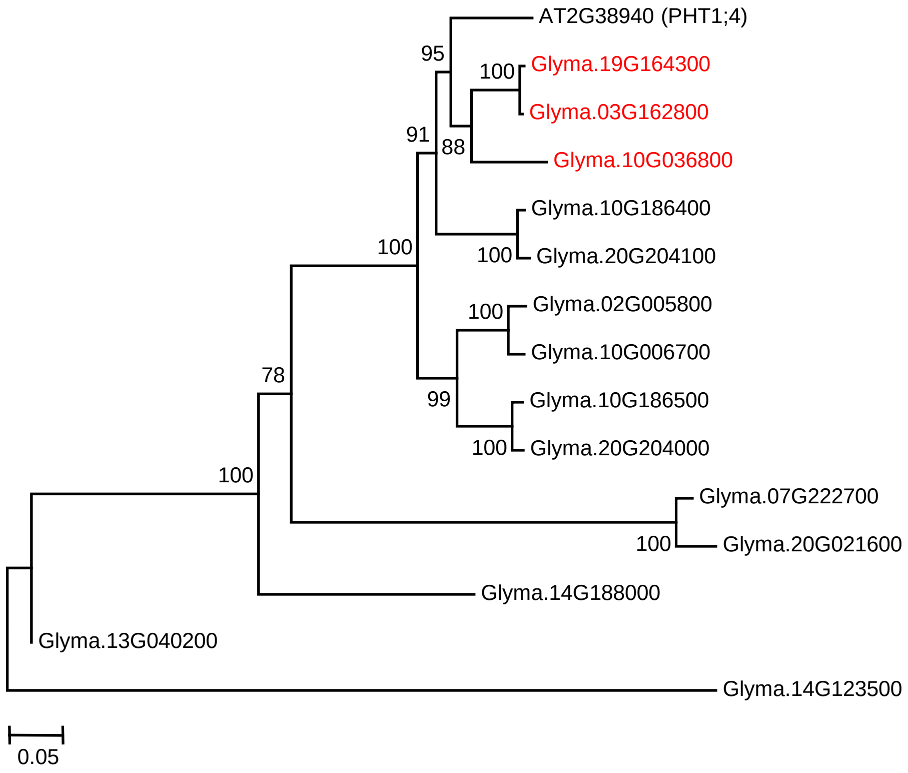

Figure S10 The NJ tree of PHT1;4-like proteins from soybean and *Arabidopsis*. The NJ phylogenetic tree was constructed based on protein sequences obtained from phytozome database (www.phytozome.net). Bootstrap values for 1,000 replicates are shown. Red indicates the nearest homology in soybean for *Arabidopsis* PHT1;4.
