# Supplementary material for: The PHOSPHATE1 genes participate in salt and Pi signaling pathways and play adaptive roles during soybean evolution
Source: BMC Plant Biol. 2019 Aug 14;19:353. doi: 10.1186/s12870-019-1959-8 (PMC6694516; doi:10.1186/s12870-019-1959-8)
Supplement: Supplementary file 2 — Table S1. Sequence identities of the GsPHO1 and GmPHO1 proteins. Table S2. Variations in the GsPHO1 and GmPHO1 proteins. Table S3. The phenotypes of GmPHO1 transgenic plants in Arabidopsis. Table S4. The germination rate of transgenic plants under 1/2 MS medium with different concentrations of NaCl. Table S5. The mRNA accumulation of PHO1 genes in roots of SN14 and ZYD6 under different stresses. Table S6. The variation pattern of PHO1 genes in response to stresses in SN14 and ZYD6. Table S7. Primers used in the present work. (PDF 535 kb) [file 12870_2019_1959_MOESM2_ESM.pdf]

The *PHOSPHATE1* genes participate in salt and Pi signaling pathways and play adaptive roles during soybean evolution

Yan Wang[1,#], Huihui Gao[1,2,#], Lingli He[1,2,#], Weiwei Zhu[1,2], Lixin Yan[1,2], Qingshan Chen[3], and Chaoying He[1,2,4,*]

1 State Key Laboratory of Systematic and Evolutionary Botany, Institute of Botany, Chinese Academy of Sciences, Nanxincun 20, Xiangshan, 100093 Beijing, China;
2 University of Chinese Academy of Sciences, Yuquan Road 19A, 100049 Beijing, China;
3 College of Agriculture, Northeast Agricultural University, 150030 Harbin, Heilongjiang, China;
4 The Innovative Academy of Seed Design, Chinese Academy of Sciences, Beijing 100101, China.
# These authors contributed equally to this work.
* Corresponding author: chaoying@ibcas.ac.cn; Tel: 86-10-62836085; Fax: 86-10-62590843

Additional file 2: Supplementary Tables (in one PDF file)
Table S1 Sequence identities of the GsPHO1 and GmPHO1 proteins.
Table S2 Variations in the GsPHO1 and GmPHO1 proteins.
Table S3 The phenotypes of *GmPHO1* transgenic plants in *Arabidopsis*.
Table S4 The germination rate of transgenic plants under 1/2 MS medium with different concentrations of NaCl.
Table S5 The mRNA accumulation of *PHO1* genes in roots of SN14 and ZYD6 under different stresses.
Table S6 The variation pattern of *PHO1* genes in response to stresses in SN14 and ZYD6.
Table S7 Primers used in the present work.

Table S1 Sequence identities of the GsPHO1 and GmPHO1 proteins.

Protein	GmPHO1;H1 (763aa)	GmPHO1;H2 (803aa)	GmPHO1;H3 (759aa)	GmPHO1;H4 (764aa)	GmPHO1;H5 (771aa)	GmPHO1;H6 (776aa)	GmPHO1;H7 (782aa)	GmPHO1;H8 (771aa)	GmPHO1;H9 (795aa)	GmPHO1;H10 (796aa)	GmPHO1;H12 (791aa)	GmPHO1;H14 (789aa)
**GsPHO1;H1 (763aa)**	**0.993**	0.290	0.316	0.938	0.771	0.318	0.269	0.762	0.309	0.301	0.449	0.447
**GsPHO1;H2 (803aa)**	0.292	**0.998**	0.506	0.297	0.296	0.506	0.874	0.299	0.723	0.714	0.306	0.310
**GsPHO1;H3 (759aa)**	0.315	0.509	**0.997**	0.317	0.310	0.914	0.479	0.314	0.533	0.523	0.313	0.319
**GsPHO1;H4 (764aa)**	0.935	0.297	0.319	**0.998**	0.777	0.320	0.277	0.767	0.313	0.306	0.457	0.454
**GsPHO1;H5 (774aa)**	0.766	0.295	0.310	0.775	**0.993**	0.314	0.279	0.953	0.319	0.312	0.461	0.456
**GsPHO1;H6 (776aa)**	0.318	0.509	0.917	0.320	0.314	**0.994**	0.479	0.316	0.533	0.526	0.311	0.317
**GsPHO1;H7 (802aa)**	0.282	0.905	0.497	0.289	0.289	0.497	**0.967**	0.291	0.715	0.704	0.300	0.303
**GsPHO1;H8 (771aa)**	0.760	0.299	0.315	0.771	0.952	0.316	0.282	**0.997**	0.324	0.317	0.463	0.459
**GsPHO1;H9 (795aa)**	0.309	0.722	0.530	0.313	0.318	0.530	0.687	0.323	**1.000**	0.902	0.311	0.313
**GsPHO1;H10 (796aa)**	0.301	0.712	0.520	0.306	0.311	0.524	0.675	0.317	0.902	**1.000**	0.309	0.311
**GsPHO1;H12 (791aa)**	0.447	0.306	0.314	0.457	0.459	0.309	0.289	0.463	0.311	0.309	**1.000**	0.947
**GsPHO1;H14 (789aa)**	0.446	0.310	0.320	0.454	0.454	0.316	0.292	0.459	0.313	0.311	0.947	**1.000**

The number in bracket indicates the amino acid length for PHO1 proteins in soybean. Bold font represents the identity of Gs-Gm orthologous pair for PHO1 protein in wild (ZYD6) and cultivated (SN14) soybeans. The red numbers indicate the range from 0.269 to 0.952 for identity between different PHO1 members.

Table S2 Variations in the GsPHO1 and GmPHO1 proteins.

GmPHO1/GsPHO1	Substitution (PROVEAN Score, prediction)	Substitution (SNAP Score, prediction)	Indels (PROVEAN Score, prediction)
PHO1;H1	I223L (-0.202, Neutral),<br>S306N (-0.741, Neutral),<br>G336E (6.662, Neutral),<br>S555G (-0.980, Neutral),<br>A581T (-1.300, Neutral).	I223L (Neutral, 93%),<br>S306N (Neutral, 97%),<br>G336E (Neutral, 72%),<br>S555G Neutral, 82%),<br>A581T (Neutral, 66%).	
PHO1;H2	Y143N (6.236, Neutral).	Y143N (Neutral, 72%).	
PHO1;H3	L99F (4.720, Neutral),<br>R600G (6.038, Neutral).	L99F (Neutral, 87%),<br>R600G (Neutral, 57%).	
PHO1;H4	V607A (-0.249, Neutral).	V607A (Neutral, 72%).	
PHO1;H5	A418V (2.807, Neutral),<br>V528I (0.508, Neutral).	A418V (Neutral, 93%),<br>V528I (Neutral, 97%).	S185_E186insVCA (-0.850, Neutral).
PHO1;H6	R53K (0.964, Neutral),<br>R274Q (1.202, Neutral),<br>M608L (1.429, Neutral),<br>K611E (3.680, Neutral).	R53K (Neutral, 97%),<br>R274Q (Neutral, 87%),<br>M608L (Neutral, 82%),<br>K611E (Neutral, 87%).	
PHO1;H7	S163F (-0.732, Neutral),<br>L322F (5.420, Neutral),<br>I363T (3.247, Neutral),<br>T523I (3.511, Neutral),<br>A579D (7.280, Neutral),<br>T603I (4.244, Neutral).	S163F (Neutral, 72%),<br>L322F (Neutral, 61%),<br>I363T (Neutral, 97%),<br>T523I (Neutral, 93%),<br>A579D (Neutral, 78%),<br>T603I (Neutral, 66%).	Y444_R445insSLF GFVVLHMLMYAA NIYFW (118.181, Neutral).
PHO1;H8	N406S (0.094, Neutral),<br>N643H (-0.364, Neutral).	N406S (Neutral, 87%),<br>N643H (Neutral, 97%).	

PROVEAN, Protein Variation Effect Analyzer (http://provean.jcvi.org.). The cutoff value of PROVEAN was -2.5, when the score more than -2.5, the prediction was neutral; when the score was less than -2.5, the prediction was deleterious. SNAP, Screening for Non-Acceptable Polymorphisms (http://www.rostlab.org/services/SNAP).

Table S3 The phenotypes of *GmPHO1* transgenic plants in *Arabidopsis.*

	Gene	Line	Germination rate (%)	The number of rosette leaves at flowering	Plant height (cm)	Seed size (mg/100 seeds)
	WT		98.15±1.5	9.75±0.46	21.71±2.33	1.46±0.15
		L2	99.07±1.0	9.70±0.47	19.44±1.47	1.50±0.08
	PHO1;H2	L8	98.15±1.5	9.80±0.88	20.50±1.81	1.36±0.13
		L9	98.15±1.5	9.60±0.98	20.08±1.26	1.53±0.12
		L15	98.10±1.5	9.67±0.49	23.52±2.40	1.55±0.03
	PHO1;H3	L16	97.22±3.0	9.89±0.32	21.63±0.94	1.61±0.17
		L18	96.27±3.0	9.83±0.39	20.07±2.56	1.40±0.10
**Class I**		L34	98.15±1.5	9.88±0.34	21.94±2.90	1.37±0.05
	PHO1;H6	L35	99.07±1.5	9.89±0.32	21.78±3.28	1.51±0.03
		L36	98.15±3.0	9.78±0.43	23.68±2.35	1.38±0.13
		L22	97.22±3.0	9.78±0.43	23.17±1.41	1.36±0.03
	PHO1;H7	L23	99.07±1.5	9.89±0.32	21.00±1.47	1.53±0.08
		L24	99.07±1.5	9.75±0.45	22.92±1.61	1.39±0.05
		L1	99.07±1.5	9.60±0.50	20.71±1.44	1.47±0.11
	PHO1;H9	L3	98.15±1.5	9.80±0.41	20.56±2.75	1.42±0.20
		L5	98.15±1.5	9.70±0.47	23.48±1.86	1.46±0.10
		L4	96.30±3.0	9.88±0.34	23.01±1.59	1.56±0.10
	PHO1;H1	L11	98.15±1.5	9.63±0.50	22.90±1.63	1.54±0.09
		L13	87.04±8.5	9.57±0.51	23.60±1.41	1.50±0.06
		L15	99.07±1.5	9.80±0.41	23.87±1.95	1.45±0.08
	PHO1;H4	L16	96.23±3.0	9.71±0.47	21.00±2.24	1.49±0.10
		L17	96.23±4.0	9.61±0.51	20.70±1.24	1.51±0.01
		L8	97.22±3.0	9.86±0.36	20.47±1.16	1.37±0.14
**Class II**	*PHO1;H5*	L11	98.15±3.0	9.70±0.47	22.90±1.77	1.43±0.06
		L14	98.15±3.0	9.60±0.50	20.90±1.17	1.40±0.15
		L7	97.22±3.0	9.80±0.41	23.38±1.41	1.44±0.05
	PHO1;H8	L8	97.22±3.0	9.70±0.47	20.88±1.15	1.38±0.06
		L10	98.15±3.0	9.90±0.31	23.46±1.62	1.50±0.17
		L2	99.07±2.5	9.80±0.41	20.16±0.78	1.49±0.17
	PHO1;H12	L12	99.83±3.0	9.78±0.43	20.71±1.20	1.38±0.03
		L16	99.07±1.5	9.86±0.36	21.99±1.45	1.52±0.06

The germination rate of transgenic plants under 1/2 MS medium for 4 days was checked. Plant height were measured for 7-week old seedlings. The number of rosette leaves were observed for flowering time.

Table S4 The germination rate of transgenic plants under 1/2 MS medium with different concentrations of NaCl.

	Gene	Line	Germination rate-2 day (%)		Germination rate-4 day (%)		Germination rate-6 day (%)	
			125 mM NaCl	175 mM NaCl	125 mM NaCl	175 mM NaCl	125 mM NaCl	175 mM NaCl
	WT		93.89±3.07	70.00±5.22	97.22±3.82	90.56±2.91	97.50±3.24	97.22±2.21
Class I	*PHO1;H2*	L2	100.00±0.00 **	83.33±1.60 **	100.00±0.00	100.00±0.00 **	100.00±0.00	100.00±0.00
		L8	100.00±0.00 **	91.67±1.29 **	100.00±0.00	97.22±1.17 **	100.00±0.00	100.00±0.00
		L9	100.00±0.00 **	91.67±1.60 **	100.00±0.00	100.00±0.00 **	100.00±0.00	100.00±0.00
	PHO1;H3	L15	100.00±0.00 **	91.67±1.43 **	100.00±0.00	100.00±0.00**	100.00±0.00	100.00±0.00
		L16	100.00±0.00**	91.67±1.29 **	100.00±0.00	97.22±1.43 **	100.00±0.00	100.00±0.00
		L18	100.00±0.00 **	88.89±1.43 **	100.00±0.00	97.22±1.17 **	100.00±0.00	100.00±0.00
	PHO1;H6	L34	97.22±2.87 *	74.44±1.29	97.22±2.46	97.22±1.43 **	97.22±1.29	100.00±0.00
		L35	97.22±1.43 *	75.93±1.43	97.22±2.46	100.00±0.00 **	100.00±0.00	100.00±0.00
		L36	100.00±0.00 **	75.00±2.87	100.00±0.00	100.00±0.00 **	100.00±0.00	100.00±0.00
	PHO1;H7	L22	100.00±0.00 **	80.56±1.60 **	100.00±0.00	97.22±1.08 **	100.00±0.00	100.00±0.00
		L23	97.22±1.60 *	83.33±1.43 **	97.22±1.43	100.00±0.00 **	100.00±0.00	100.00±0.00
		L24	100.00±0.00 **	91.67±1.60 **	100.00±0.00	100.00±0.00 **	100.00±0.00	100.00±0.00
	PHO1;H9	L1	100.00±0.00 **	94.44±1.17 **	100.00±0.00	97.22±1.29 **	100.00±0.00	100.00±0.00
		L3	100.00±0.00 **	94.44±1.43 **	100.00±0.00	100.00±0.00 **	100.00±0.00	100.00±0.00
		L5	100.00±0.00 **	91.67±1.17 **	100.00±0.00	100.00±0.00 **	100.00±0.00	100.00±0.00
Class II	*PHO1;H1*	L4	97.22±1.60 *	91.67±1.43 **	100.00±0.00	100.00±0.00 **	100.00±0.00	100.00±0.00
		L11	99.07±0.0143 *	88.89±1.29 **	100.00±0.00	97.22±1.17 **	100.00±0.00	100.00±0.00
		L13	100.00±0.00 **	94.44±2.19 **	100.00±0.00	100.00±0.00 **	100.00±0.00	100.00±0.00
	PHO1;H4	L15	100.00±0.00 **	75.00±1.60	100.00±0.00	97.22±1.29 **	100.00±0.00	100.00±0.00
		L16	100.00±0.00 **	75.93±1.43	100.00±0.00	97.22±1.29 **	100.00±0.00	100.00±0.00
		L17	100.00±0.00 **	76.85±1.29	100.00±0.00	100.00±0.00 **	100.00±0.00	100.00±0.00
	PHO1;H5	L8	97.22±1.60 *	91.67±1.43 **	100.00±0.00	100.00±0.00 **	100.00±0.00	100.00±0.00
		L11	97.22±1.43 *	94.44±2.19 **	100.00±0.00	100.00±0.00 **	100.00±0.00	100.00±0.00
		L14	100.00±0.00 **	91.67±2.87 **	100.00±0.00	97.22±1.29 **	100.00±0.00	97.22±1.17
	PHO1;H8	L7	100.00±0.00 **	91.67±1.43 **	100.00±0.00	94.44±1.29 *	100.00±0.00	97.22±1.01
		L8	97.22±3.21 *	88.89±1.43 **	100.00±0.00	97.22±1.08 *	100.00±0.00	97.22±1.08
		L10	100.00±0.00 **	91.67±1.43 **	100.00±0.00	94.44±1.17 *	100.00±0.00	97.22±1.01
	PHO1;H12	L2	97.22±2.48 *	94.44±1.08 **	97.22±1.60	100.00±0.00 **	97.22±1.29	100.00±0.00
		L12	100.00±0.00 **	91.67±1.17 **	100.00±0.00	97.22±1.29 **	100.00±0.00	100.00±0.00
		L16	100.00±0.00 **	91.67±1.43 **	100.00±0.00	100.00±0.00 **	100.00±0.00	100.00±0.00

Seeds were planted on 1/2MS medium with different concentration of NaCl. Seed germination rate was recorded with the radical penetrating from the seed coat at 2 days, 4 days and 6 days after stratification. The experiments were performed using three independent biological samples. The difference between wild type and transgenic lines were checked by two-tailed student's *t*-test. Bar=1.5 cm. The * means significance at a $P < 0.05$ level, and the ** represent the significance at a $P < 0.01$ level.

Table S5 The mRNA accumulation of *PHO1* genes in roots of SN14 and ZYD6 under different stresses.

	Gene	Salt-stress					Pi-stress				
		Relative ratio	The relative expression				Relative ratio	The relative expression			
			SN14-normal	SN14-stess	ZYD6-normal	ZYD6-stress		SN14-normal	SN14-stess	ZYD6-normal	ZYD6-stress
Class I	*PHO1;H2*	0.4033	0.4033	0.4697	0.4796	0.7122	0.1684	0.4364	0.3934	0.3092	0.4445
	PHO1;H3	0.0548	0.0548	0.1237	0.0718	0.0804	1.9185	17.9239	19.7460	19.0349	22.0593
	PHO1;H6	0.5396	0.5396	0.6352	0.4965	0.6479	3.8906	41.3299	44.6980	40.5364	48.2622
	PHO1;H7	0.0775	0.0775	0.3766	0.1224	1.2352	0.0114	0.2412	0.0908	0.3945	0.0996
	PHO1;H9/10	2.4794	2.4794	5.3158	4.5948	9.7127	0.0791	30.3837	7.9478	21.8296	22.6466
Class II	*PHO1;H1/H4*	6.8211	6.8211	5.6044	4.9588	1.9709	2.5491	19.1991	24.3520	11.4033	21.4565
	PHO1;H5	4.2871	4.2871	3.2448	9.0631	5.2556	0.1387	1.4929	1.1341	0.9821	1.4814
	PHO1;H8	1.4241	1.4241	2.5585	1.7900	2.9935	1.0000	9.9288	7.0671	4.5246	6.3755
	PHO1;H12/H14	1.0000	1.0000	4.5553	1.0140	7.5389	0.0759	0.9915	4.0459	0.5267	4.1394

The relative expression of each gene was evaluated using the mean of the expression data under the corresponding conditions. The expression of *PHO1;H12/H14* in salt stress while the expression *of PHO1;H8* in low-Pi was set as 1. Detailed data were available in Figures 3 and 4. Since the Class II genes were grouped with *AtPHO1;H1* and *AtPHO1* (Figure S2), and they were predominantly expressed in roots (Figures 2 and S3), the Class II genes might be likely the major players in this gene family involving in stress responses, as confirmed by transgenic *Arabidopsis* analysis.

Table S6 The variation pattern of *PHO1* genes in response to stresses in SN14 and ZYD6.

	Gene	Salt-stress			Pi-stress		
		Ratio (stress/normal) SN14	Ratio (stress/normal) ZYD6	Pattern	Ratio (stress/normal) SN14	Ratio (stress/normal) ZYD6	Pattern
Class I	*PHO1;H2*	1.1647	1.4850	S3	0.7250	1.3370	P3
	PHO1;H3	2.2575	1.1201	S4	1.3097	2.3041	P1
	PHO1;H6	1.1771	1.3047	S1	1.0520	3.0255	P4
	PHO1;H7	4.8601	10.0892	S1	0.8437	0.8592	P2
	PHO1;H9/10	2.1440	2.1139	S1	0.6185	1.8545	P3
Class II	*PHO1;H1/H4*	0.8216	0.3974	S2	1.7663	2.6685	P1
	PHO1;H5	0.7569	0.5799	S2	0.8230	1.9529	P3
	PHO1;H8	1.7966	1.6723	S1	1.0408	1.8582	P4
	PHO1;H12/H14	4.5553	7.4351	S1	3.4823	9.6356	P1

The response of each gene to the stresses was evaluated by the mean of the ratio of the expression data under the stressed conditions to the corresponding controlled ones. Overall, four patterns of gene expression variation were defined under salinity stresses (S1-S4), and Pi-deficiency conditions (P1-P4) among wild and cultivated soybeans.

Table S7 Primers used in the present work.

Gene	Forward (5'-3')	Reverse (5'-3')	
SOS2	ATTGAGGCTGTAGCGAAC	GGTATTCCTTCTGTTGCC	qRT-PCR
SOS3	GGAGGAATCTCTTCGCTG	CACGAAAGCCTTATCCACC	
FRY1	CGCAGTAGCACTAGGATTG	TTGACACCGAGTTTATTGG	
P5SC1	TTCTCAGATGGTTTCCAGGTTG	TGGGAATGTCCTGATGGGTG	
ADH1	CTCTTGGTGCTGTTGGTTTAGG	AATTGGCTTGTCATGGTCTTTC	
AT4	GAGCGATGAAGATTGCATGAAG	GATCGAAGTTGCCCAAACGA	
IPS1	GGGATGGCCTAAATACAAAATGAA	TCCATATCTTAAAACGCTTTCCTTACA	
PHO1;H1	TACCGATTGGAGAATGAGCATCTAA	TTAGTCTTCTTCATCCACTTCTCTGAAAG	
PHT1;4	TGTGCCGGCCGAAATCT	TTGCTCCTAATTTTCCTGATGCT	
H01	CGGGTCGACAGATGGTGAAATTCTCAAAGGAG	GGCATTTAAATGGCTGTCTGAGTCTATGTCACGAAA	transgenic constructs
H02	CGGGTCGACAGATGAAGTTTGGCAAGGAATTTGCTG	GGCATTTAAATGCTCATCCTTATCTTCCTCCTCCTCG	
H03	CGGGTCGACAGATGAAGTTTGGGAAAGAATTTG	GCCATTTAAATGTATGACTGTATTCTCTTCGTCATCTTC	
H04	CACGTCGACAGATGGTGAAATTCTCGAAGGAGCTCG	GGTATTTAAATGGCTGTCTGAGTCTATGTCACGAAATG	
H05	CGGGTCGACAGATGGTGAAGTTCTCAAAGGAGCTA	GGCATTTAAATGGTCAGAGTCTATCTCTCGAAATGG	
H06	CGGGTCGACAGATGAAGTTTGGGAAAGAATTTG	GCCATTTAAATGTATGACTGTATTCTCTTCCTCATCTTC	
H07	CGGGTCGACAGATGAAGTTTGGCAAGGAATTTGCTGC	GCCATTTAAATGCTCATCCTTATCTTCCTCTTCGTCG	
H08	CGGGTCGACAGATGGTGAAGTTCTCAAAGGAGCTAGA	GGCATTTAAATGGTCAGAGTCTACCTCTCGAAATGG	
H09	CGGGTCGACAGATGAAGTTTGGGAAAGAATACACA	GGCATTTAAATGCTCATCTTTATCTTCATCTTCGTC	
H12	CGGGTCGACAGATGGTGAAGTTCTCAAAGCA	GGCATTTAAATGGTCTTCTTCATCCATTTCAT	

Primers for phosphate-deficiency marker genes in *Arabidopsis*, such as *IPS1*, *AT4*, *PHO1;H1* and *PHT1;4*, were previously described [3]. Primers for salt-tolerance pathway genes in *Arabidopsis* were previously described [56]. The primers used for cDNA isolation and qRT-PCR analyses of soybean *PHO1* genes are available in the previous report [45].
